# Effect of Multimodal App-Based Interventions on Glycemic Control in Patients With Type 2 Diabetes: Systematic Review and Meta-Analysis

**DOI:** 10.2196/54324

**Published:** 2025-01-24

**Authors:** Emma Bodner, Lena Roth, Kathleen Wiencke, Christian Bischoff, Peter EH Schwarz

**Affiliations:** 1 Sidekick Health Germany GmbH Hamburg Germany; 2 Department for Prevention and Care of Diabetes Faculty of Medicine Carl Gustav Carus Technische Universität Dresden Dresden Germany; 3 Department of Neurology Max Planck Institute for Human Cognitive and Brain Sciences Leipzig Germany

**Keywords:** app-based interventions, diabetes mellitus type 2, digital health, real world evidence, pragmatic trials, ehealth, mhealth, HbA_1c_, systematic review, meta-analysis, interventions, app, glycemic, patients, type 2 diabetes

## Abstract

**Background:**

Digital technologies for type 2 diabetes mellitus (T2DM) care hold great potential to improve patients’ health in the long term. Only a subset of telemedicine offerings are digital interventions that meet the criteria for prescribable digitale Gesundheitsanwendung (digital health apps; DiGAs) in Germany. Digital treatments further provide vast amounts of patient data that are important to generate evidence.

**Objective:**

This systematic review aims to analyze the efficacy of multimodal digital therapies that mainly meet the DiGA criteria for patients with T2DM and to elicit the potential of such therapies. This includes evidence from randomized controlled trials (RCTs) as well as from real-world data. The outcome of interest was a reduction in glycated hemoglobin (hemoglobin A_1c_ [HbA_1c_]; long-term blood glucose measurements).

**Methods:**

A systematic literature search was conducted in the literature bases PubMed, LIVIVO, and Cochrane, based on the predefined PICO (Population; Intervention; Control; Outcome) scheme. Identified studies were assessed for risk of bias, pragmatism, and overall quality of evidence. Meta-analyses were conducted for between group differences using RCTs only, and for within-group differences using RCTs and non-RCTs, to examine the effect of the interventions on HbA_1c_.

**Results:**

In total, 795 records were identified, of which 24 were eligible for this systematic review and 23 studies were eligible for the meta-analysis. The results of the meta-analyses showed significant and clinically relevant reductions in HbA_1c_ in patients with T2DM. Regarding the between-group difference for HbA_1c_ reduction, the pooled effect of the RCTs showed a reduction of –0.36% (95% CI –0.59% to –0.14%; *P*<.001), favoring app-based interventions. The average mean within-group reduction in HbA_1c_ was –0.79 (95% CI –1.02 to –0.55), with no significant difference between RCTs (–0.69, 95% CI –1.13 to –0.24) and non-RCTs (–0.87, 95% CI –1.16 to –0.57; *P*<.01, differences between RCTs and RCTs *P*=.44). A pragmatism rating showed that both study types were on average (very) pragmatic, that is, close to usual care. However, the overall quality of evidence was low to very low.

**Conclusions:**

This systematic review shows that digital therapies that mainly meet the DiGA criteria can effectively improve HbA_1c_ in patients with T2DM. The integration of digital health care into usual care holds great potential and should be considered as a complementary option to usual care in the future.

**Trial Registration:**

PROSPERO CRD42023440203; https://www.crd.york.ac.uk/prospero/display_record.php?RecordID=440203

## Introduction

### Background

Type 2 diabetes mellitus (T2DM) is one of the leading causes of death as well as disability-adjusted life years [1]. The rapidly rising global prevalence of T2DM in adults, especially in rural and high-income areas, was estimated to be 10.5% in 2021 [2]. Those levels were previously estimated to occur in 2030 [3]. The metabolic disease is characterized by elevated blood glucose levels, which are associated with an increased risk for vascular and cardiovascular complications, such as heart disease, chronic kidney disease, diabetic retinopathy, and diabetic foot ulcers [4,5]. In addition, T2DM is related to the occurrence of mental health disorders, such as depression [6]. This causes T2DM to be associated with an increased mortality rate in patients enduring the disease [4,7]. Consequently, the chronic condition poses a major health burden on the physical and mental health of those affected as well as on their families [3].

There are several known risk factors for developing T2DM. In particular, conditions such as obesity, high blood pressure, and hyperlipidemia, are associated with relevant lifestyle factors such as high-caloric diets, sedentary behavior, smoking, and alcohol consumption [8,9]. To counteract the multiple risk factors of the disease, guidelines describe lifestyle modification measures as the foundation of all therapeutic interventions in patients with T2DM [10]. Behavioral interventions toward a healthy lifestyle play an important role in preventing the onset of T2DM in individuals at high risk and decreasing the development and progression of diabetic complications for patients with manifested T2DM [11].

### Multimodal Therapy

A multimodal therapeutic approach for people with obesity and T2DM combines nutritional medical intervention, exercise, and behavioral therapy support and addresses treatment adherence to enhance the long-term success of such interventions [12]. These components of interdisciplinary treatment can be communicated to patients digitally. For example, eating behavior can be tracked and then a reduction in calorie intake and optimization of food composition can be implemented using app features. In addition, training sessions or tasks to increase physical activity can also be integrated. To increase therapy engagement, interventions should not only be multidisciplinary but also patient-centered and address as many risk factors as possible [5,11].

In line with that, behavioral interventions can also reduce the pharmacotherapy escalation. In a trial applying a multimodal approach, including nutritional and behavioral counseling, educational content, digital coaching, and medication management, most participants, 42.7%, decreased their medication, while another 8% eliminated their medications. Hemoglobin A_1c_ (HbA_1c_; long-term blood glucose) levels were also significantly reduced with 56.1% of participants achieving a HbA_1c_ value of <6.5% [13].

Blood glucose control is a key target for such interventions, to lower cardiovascular risk as well as mortality from T2DM [5]. In adults with T2DM, the HbA_1c_ target value is set individually, depending on various factors, such as diabetes duration, comorbidities, and patient preference, but it lies within the corridor of 6.5%-8.5% [10]. Accordingly, guidelines for the treatment of T2DM formulate lifestyle interventions to manage indicators such as weight, blood pressure, and blood lipids as the foundation of the prevention and treatment of T2DM and also promote self-management and patient autonomy as a therapeutic goal [10,14]. Due to the chronic nature of T2DM, patients must manage their disease in their daily lives independently of medical care [15].

### Digital Therapy

In this context, digital technologies, for example, tracking and providing a feedback loop with health care professionals (HCPs) are already included in guidelines as having great potential to improve diabetes care [5,16].

With the Digital Healthcare Act (January 2020) Germany was the first country to use a legal health claim for insured individuals to receive evidence-based treatment in the form of digitale Gesundheitsanwendung (digital health apps [DiGA]), that is, apps prescribed by health care providers [17]. Generally, DiGAs are not primary prevention tools but support the monitoring, discovery, and treatment of diseases, injuries, and disabilities. Other countries, in particular France and Belgium, are following the example and pursuing easier market access for digital solutions in the health care sector [18].

The potential of digital interventions to reduce HbA_1c_ levels has been shown in previous systematic reviews [19-21]. For instance, one umbrella review reports mean reductions in patients with T2DM using telehealth interventions of between −0.01% and −1.13% [21]. The medical purpose of a DiGA is achieved through the interaction of the patient with digital technology [22]. This means that DiGAs are not purely digital communication channels between patients and HCPs, but treatment is largely carried out independently by the patient through an app [20,22]. This means that therapy with DiGAs is independent of the prescription site in which it is used, as the driver of the therapeutic effect is the patient’s interaction with the application. By focusing on apps that meet the definition of DiGA, we aim to analyze the evidence for these technologies in diabetes care. This also allows us to expand the current evidence base for the digitalization of usual care, for example, delivering care through phone calls or chats.

### Evidence Based on Different Study Designs

For a medical device to be approved as a DiGA in Germany, its effectiveness has to be shown with clinical evidence generated by randomized controlled trials (RCTs) [23]. Especially before approval or after a device is already approved and on the market, digital applications, including DiGAs, offer new opportunities to generate evidence based on vast amounts of actual user and patient data. In this context, real-world data (RWD) and real-world evidence are increasingly recognized as complementary to RCTs. For example, user data allows us to continuously investigate the efficacy after approval as part of postmarket research, offering additional insights into how medical devices perform [24]. The European Medicines Agency, defines RWD as “routinely collected data relating to a patient’s health status or the delivery of health care from a variety of sources other than traditional clinical trials” [25]. One type of such data is health records directly tracked in-app. Evidence generated with RWD is often considered pragmatic and externally valid, making it cost- and time-efficient, particularly for regulatory purposes, such as postmarket surveillance under the European Medical Device Regulation [23,24,26,27].

RCTs provide high internal validity by controlling for confounding variables and minimizing bias through more controlled circumstances and more rigid protocols. Non-RCTs contribute by offering evidence generated outside a study setting and, while less controlled, still provide valuable information on how interventions perform in the real world. Both approaches offer opportunities for implementation in pragmatic settings [28]. To better distinguish between the internal and external validity of trials and the potential balance between them, we use both the GRADE (Grading of Recommendations, Assessment, Development and Evaluation) and Pragmatic Explanatory Continuum Indicator Summary (PRECIS-2) tools, respectively.

### Research Question

Current evidence on digital therapies exists for a broad range of intervention types but not specifically for apps that meet the definition of a DiGA addressing T2DM. The objective of this systematic review was to analyze the efficacy of such and to elicit their potential, including explanatory as well as pragmatic studies.

## Methods

### Search Strategy and Eligibility Criteria

A systematic literature search was conducted in January 2023 to identify evidence on the efficacy of multimodal, app-based lifestyle interventions that meet the definition of DiGAs in reducing HbA_1c_ in patients with T2DM. The search was performed in the electronic databases PubMed, LIVIVO, and Cochrane and the search strategy was individually tailored for each database. A combination of search terms, MeSH (Medical Subject Headings) terms, and appropriate Boolean operators was developed (Multimedia Appendix 1) based on the PICO (Population; Intervention; Control; Outcome) scheme (Table 1) to identify relevant studies. To generate a broader evidence base, not only RCTs but also other study designs were considered eligible (eg, observational studies or analyzing user data). Study selection was carried out per the PRISMA (Preferred Reporting Items for Systematic Reviews and Meta-Analyses) guidelines [29] and the title-abstract screening and full-text screening of the search results were performed using CADIMA [30]. The screening was independently conducted by EB and LR, and disagreements were resolved through discussion and consensus, involving a third party if necessary.

**Table 1:** Inclusion and exclusion criteria according to the PICO^a^-scheme.

	Inclusion	Exclusion
Population	Adult patients with diagnosed diabetes mellitus type 2 and HbA_1c_^b^ ≥6.5%	Age <18 years, type 1 diabetes, gestational diabetes, or focus on special subgroups of populations (eg, low income)
Intervention	App-based lifestyle interventions meeting the digitale Gesundheitsanwendung (digital health app) definition, that is, primarily stand-alone (additional human support was possible, if not as the main driver) or multimodal, that is, covering at least two areas of diabetes care (diet, exercise, self-management, etc)	Interventions not primarily app-based (ie, the need for human resources to deliver intervention) or not multimodal (ie, covering only one area of diabetes care, eg, diet intervention), pharmacological interventions, or interventions for prevention
Control	All kinds of control groups or no control group	N/A^c^
Outcome	Glycemic control (HbA_1c_)	HbA_1c_ not reported
Study design	Systematic reviews^d^, meta-analyses^d^, randomized controlled trials, observational studies, or pilot studies	Study protocols, case reports, surveys, qualitative studies, narrative literature reviews, cross-sectional studies, scoping reviews, economic analyses, or books

^a^PICO: Population, Intervention, Control, Outcome.

^b^HbA_1c_: hemoglobin A_1c_ (long-term blood glucose).

^c^Not applicable as control was not defined as an exclusion criteria.

^d^Identified systematic reviews were screened for studies that met the inclusion criteria of the present review.

### Grading of Pragmatism (PRECIS-2)

Both RCTs and non-RCTs were graded by EB, KW, and LR using the PRECIS-2 tool [31] to represent how explanatory or pragmatic the trials were. The PRECIS-2 tool covers nine domains: “eligibility,” “recruitment,” “organization,” “setting,” “flexibility (adherence),” “flexibility (delivery),” “follow-up,” “primary endpoint,” and “primary analysis.” All domains are rated on a 5-point Likert scale from 0 (very explanatory) to 5 (very pragmatic). Generally, a trial is more pragmatic the less strict its protocol is or the fewer additional resources are used, and is therefore closer to usual care. On the other hand, a very explanatory trial would differ vastly from the usual care setting, using strict protocols, a lot of extra resources, specific patients as well as study settings or collecting a vast amount of extra data.

To grade the pragmatism of the studies included in this systematic review, a mean over all domains for each study and across all studies within the respective study type (RCT vs non-RCT) was calculated and was used to compare pragmatism between domains, studies, and study types.

### Grading of Evidence (GRADE) and Risk of Bias Assessment

Risk of bias (RoB) assessments for the included studies were independently performed by 2 reviewers, and disagreements were resolved through discussion and consensus. The RCTs were assessed by CB and EB using the RoB 2 tool [32] and nonrandomized and observational studies were assessed by EB and LR, using the Risk Of Bias in Non-Randomized Studies of Interventions (ROBINS-I) tool [33]. The quality of evidence was evaluated by EB, KW, and LR using the GRADE tool [34]. This established tool was used to assess the overall certainty of evidence according to RoB, imprecision, inconsistency, indirectness, and publication bias (see Multimedia Appendix 2) [34]. This was carried out using the GRADEpro Guideline Development Tool (GRADEpro GDT) for the three outcomes: changes in HbA_1c_ between groups in RCTs, within groups in RCTs, and within groups in non-RCTs. In addition, publication bias was evaluated with the help of funnel plots using the R (R Foundation) function *meta::forest.meta* [35].

### Statistical Analysis

The data analysis, that is, pooling the results, was performed with R (version 4.3.0) in the RStudio environment.

Two approaches were used to pool the results of the studies. First, the unstandardized between group differences were used to assess the effect of the intervention group (IG) or unit compared with the control group (CG) or unit. Second, with the within-group mean differences (MDs) the absolute change in HbA_1c_ postintervention was quantified. Both effect sizes were not standardized because the included studies measured the relevant outcome on the same scale, that is, HbA_1c_ (in percentage) [36]. The results (mean [SD]) with the highest statistical quality were extracted: (1) for RCTs intention-to-treat (ITTs) analyses that analyze all participants as randomized independent of protocol adherence (with imputation or complete cases analysis) were preferred over per-protocol analyses including only patients adhering to the protocol and hence hold the potential of biased “best case” results and (2) generally adjusted values were preferred over the potentially confounded.

While *meta::metacont* can calculate the mean between group difference and the respective 95% CIs based on each within-group MD and corresponding SD, the within-group MD and corresponding SE needed to be precalculated before effects could be pooled with *meta::metagen* [37,38]. The function further allowed us to calculate prediction intervals for the expected intervention effect in a single new study [39]. Random-effects models were used to pool results because study heterogeneity cannot be excluded (ie, due to different observation periods and different digital apps). Missing within-group MDs and SDs for the IG (and CG) were calculated from the available information reported in the studies per the following suggested procedures. MDs between IG and CG were calculated based on the formula for within-group differences, that is, the mean of the follow-up value subtracted by the mean of the baseline value [36]. In case 95% CIs, *t* statistic values, or *P* values were available, the SD was calculated based on the formulas provided by Higgins et al [40]. If only baseline or follow-up SDs were available, one was substituted with the other; under the assumption that the intervention did not alter the variance [40]. Assuming that the correlation coefficient between the pre- and postvalue of the IGs was similar, missing SDs for the within-group differences were calculated by the mean of the baseline and follow-up SDs [40].

For heterogeneity assessment, the *I*^2^ statistic was used. It quantifies the amount of variation in the results that is not random. Generally, an *I*^2^ below 40% can be considered low, an *I*^2^ between 30%-60% moderate, and an *I*^2^ between 50%-70% or even 70%-90% high [41]. In case of high heterogeneity outlier analysis based on the study by Harrer et al [42] was conducted and reported.

## Results

### Study Selection

**Figure 1:**
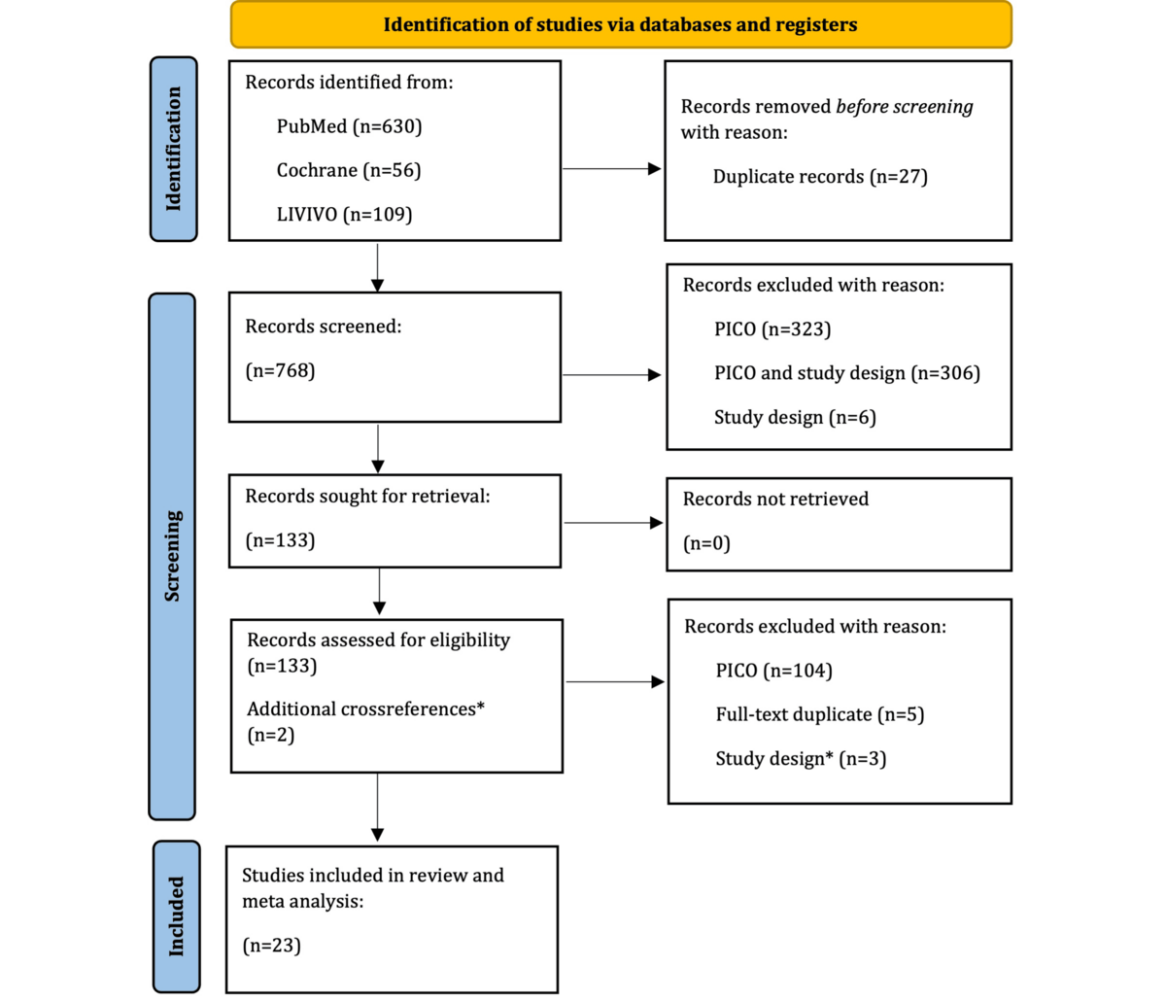
PRISMA flow chart of included and excluded studies within this systematic review and meta-analysis. *The content of identified systematic reviews (n=3) was screened to identify further eligible trials that were included as crossreferences. Systematic Reviews were not included in this study. PICO: Population; Intervention; Control; Outcome; PRISMA: Preferred Reporting Items for Systematic Reviews and Meta-Analyses.

Overall, 21 trials and 3 systematic reviews met the inclusion criteria and were found to be eligible. Systematic reviews were not included in this review and meta-analysis but were used to identify further eligible trials. Their content was screened and thereby, two further trials were identified and included as cross-references. In total, 23 studies were included in this systematic review and were eligible for meta-analysis (see PRISMA flow diagram in Figure 1 and checklist in Multimedia Appendix 3).

### Characteristics of Included Studies

A total of 23 studies were eligible for the review, 12 RCTs and 11 non-RCTs. Of those final studies, 3 were performed as secondary analyses looking at specific subgroups from another study (Lee et al 2021 [43] and Lim et al 2022 [44]) or presented preliminary results of a trial (Torbjørnsen et al 2014 [45]). They were hence not included in the meta-analysis because the main results are covered by other included studies. Another study (Dixon et al 2020 [46]) only looked at HbA_1c_ changes based on different HbA_1c_ baseline categories and was also not included in the meta-analysis because a result for the whole sample could not be obtained and including several subgroups would overrepresent the single study.

Overall, nine of the studies included in the meta-analysis were RCTs and 10 were non-RCTs. Most of the non-RCTs were single-arm observational (pilot) studies and two had an intraindividual CG (Table 2). The average calculated pragmatism of each study is reported in Table 2, while detailed information on each domain is provided in Multimedia Appendix 4. Detailed information on the content and the features of the apps is provided in Multimedia Appendix 5. Most RCTs were performed in Asia, most non-RCTs were performed in the United States. The patient characteristics of study participants and app users are provided in Table 3.

**Table 2:** Overview of studies included in the systematic review and meta-analysis.

Reference		PRECIS-2^a,b^	Intervention	Intervention duration	Country
**RCTs^c^**					
	[47]	4.3	Sidekick Health (Sidekick Health)	6 months	Iceland
	[45,48]	4.4	Few Touch Application (owned by the Norwegian Centre for Integrated Care and Telemedicin)	12 months	Norway
	[43,49]	3.7	Switch (Huraypositive Inc)	6 months	South Korea
	[50]	4.4	LIBIT (Huraypositiv) + Medilarm (GST Korea)	3 months	South Korea
	[51]	4	iCareD (Medical Excellence Inc)	26 weeks	South Korea
	[44,52]	3.8	nBuddy Diabetes (HeartVoice)	6 months	Singapore
	[53]	4.3	N/A^d^ (developed for research)	12 weeks	Indonesia
	[54]	4.2	DialBetics (Department of Ubiquitous Health Informatics, NTT DOCOMO)	12 weeks	Japan (Tokyo)
	[55]	4.7	N/A (developed for research)	6 months	China
**Non-RCTs**					
	[56]^e^	4.3	Wellthy CARE (Wellthy Therapeutics Pvt Ltd)	4 months	India
	[57]^f^	4.3	Time2Focus (Focus-Complementary Medicine)	12 weeks	United States
	[58]^e^	4.3	FareWell (Better Therapeutics LLC)	12 weeks	United States
	[59]^g^	4.3	Vitadio (Vitadio Health)	3 months	Germany
	[46]^h^	4.9	Onduo Virtual Diabetes Clinic (Verily Life Sciences LLC)	N/A	United States
	[60]^h^	4.9	BlueStar (WellDoc Inc)	N/A	Mainly the United States
	[61]^f^	4.3	N/A	12 week	South Korea
	[62]^e^	4.1	GlycoLeap (Holmusk, Inc)	6 months	Singapore
	[63]^e^	4.4	Onduo Virtual Diabetes Clinic	4 months	United States
	[64]^e^	4.4	Vida Health (Vida Health)	N/A	United States
	[65]^g^	4.7	Vida Health (Vida Health)	N/A	Mainly the United States

^a^The Pragmatic Explanatory Continuum Indicator Summary tool was used to rate pragmatism. Numbers are the mean over all domains per study. The highest grade (5) indicates a very pragmatic trial and the lowest grade (1) indicates a very explanatory trial.

^b^PRECIS-2: Pragmatic Explanatory Continuum Indicator Summary.

^c^RCT: randomized controlled trial.

^d^N/A: not (publicly) available.

^e^Observational study.

^f^Observational pilot study.

^g^Intraindividual observational study.

^h^Analysis of user data.

**Table 3:** Patient characteristics^a^.

Reference			n/N in analysis^b^		Dropout, n (%)		Baseline HbA_1c_ (%), mean (SD)		Age (year), mean (SD)	Gender female (%)		Disease duration (year), mean (SD)		Medication^c^
**RCTs^d^**														
	[47]	30/37		IG^e^: 3 (17); CG^f^: 4 (21)		IG: 7.7 (2); CG: 7.8 (1.9)		51.2 (10.6)		62	IG: 4.9 (5.1); CG: 7.4 (4.4)		O/I^g^	
	[45,48]	IG: 80/101; CG: 79/101		IG: 11 (24); IG+HCP^h^: 10 (20); CG: 8 (18)		IG: 8.1 (1.1); IG+HCP: 8.2 (1.1); CG: 8.3 (1.2)		57 (12)		41	IG: 11.2 (7.3); IG + HCP: 9.6 (8.4); CG: 9.4 (5.5)		O/I	
	[43,49]	IG: 136/148; CG: N/A^i^/72		IG: 10 (13.5); CG: 2 (2.7)		IG: 8.2 (1.5); CG: 8 (1.2)		IG: 51.4 (7.9); CG: 52.6 (7.9)		IG: 42; CG: 31	IG: 7.5 (4.8); CG: 9.3 (6)		O/I	
	[50]	48/50		IG: 2 (8); CG: 0 (0)		IG: 7.4 (0.6); CG: 7.6 (0.8)		IG: 56 (8.1); CG: 63 (8.5)		IG: 56; CG: 56	IG: 7.9 (6.3); CG: 10.8 (8)		O/I	
	[51]	269/269		IG: 12 (13.2); IG+HCP: 7 ( 7.7); CG: 16 (18.4)		IG: 8.7 (1.3); IG + HCP: 8.8 (1.4); CG: 8.6 (1.1)		IG: 51.3 (13.1); IG + HCP: 53.6 (11.7); CG: 52.6 (12.1)		IG: 56; IG + HCP: 58; CG: 57	IG: 10.9 (8.3); IG + HCP: 11.9 (7.8); CG: 11.5 (8.2)		O/I	
	[44,52]	IG: 204/204; CG: N/A/171		IG: 5 (5.1); CG: 4 (3.8)		IG: 7.4 (1.2); CG: 7.5 (1.3)		IG: 51.6 (9.4); CG: 50.8 (10)		IG: 33.3; CG: 37.1	IG: 5.2 (4.5); CG: 4.2 (3.6)		O^j^	
	[53]	60/60		0		IG: 8 (2); CG: 8.6 (3)		IG: 56.2 (7.63); CG: 54.5 (9.2)		IG: 80; CG: 63	N/A		N/A	
	[54]	54/54		IG: 3 (11.1); CG: 2 (7.4)		IG: 7.1 (1); CG: 7 (0.9)		IG: 57.1 (10.2); CG: 57.4 (9.4)		IG: 26; CG: 22	IG: 9.6 (7); CG: 8.5 (8)		O/I	
	[55]	120/120		0		IG: 8.6 (2.3); CG: 8.7 (2.3)		IG: 45.1 (8.7); CG: 45.8 (8.4)		IG: 45; CG: 48	N/A		O/I	
**Non-RCTs**														
	[56]	102/102		N/A		8.5		50.8		31	N/A		N/A	
	[57]	201/201		N/A		IG: 9 (1.2); CG: 9 (1.2)		IG: 56.1 (11); CG: 58.5 (10.8)		IG. 49; CG: 53	N/A		O/I	
	[58]	97/118		9 (7.6)		8.1 (1.6)		50.7 (9.4)		81	2.6 (1.6)		1.4 (0.9)^k^	
	[59]	42/60		18 (30)		IG: 7.9 (1.0); CG: 8.2 (1.3)		57 (7.4)		45	7.6 (6.4)		7.7 (1.7) ^k^	
	[46]	N/A/740		N/A		7.7 (1.7)		53.8 (8.8)		63	N/A		7.7 (1.7) ^k^	
	[60]	372/3142		N/A		N/A		N/A		50	N/A		O/I	
	[61]	29/29		N/A		7.7 (0.7)		53.9 (9.1)		31	N/A		O/I	
	[62]	83/100		17 (17)		8.8 (1.6)		53.5 (9.6)		50	9.3 (7.3)		O	
	[63]	55/60		5 (8.3)		8.9 (1)		57.3 (11.6)		40	N/A		O/I	
	[64]	1128/1934		N/A		9.8 (1.7)		54.1 (10)		65	N/A		N/A	
	[65]	211/950		78^l^		8.8 (1.6)		59.3 (11.3)		59	N/A		N/A	

^a^Patient characteristics are described on the initially included patients at baseline (when available). However, information was not always available for the whole sample (eg, medication or duration of disease). When possible, sample characteristics were provided for the intervention groups and control groups separately.

^b^n refers to the patients included in the analysis whose result is used for the meta-analysis and does not necessarily correspond to the sample after dropout.

^c^Medication is provided as reported in the studies, either as the number of medications (mean [SD]) or the type of medication.

^d^RCT: randomized controlled trial.

^e^IG: intervention group.

^f^CG: control group.

^g^O/I: oral and insulin or a mix of both.

^h^HCP: health care professionals.

^i^N/A: not applicable.

^j^O: oral medication only.

^k^Mean (SD).

^l^Drop out for analysis of real-world users was defined as the users initially included in this study but excluded for the analysis due to missing (follow-up) HbA_1c_ values.

### Grading of Pragmatism

To rate the level of pragmatism of the included studies, the PRECIS-2 tool was used (Multimedia Appendix 4). The mean score of pragmatism for RCTs was 4.2 and for non-RCTs 4.5, that is, both study types were on average (very) pragmatic, that is, close to usual care. As a routinely collected parameter in usual care, the HbA_1c_, is a very pragmatic parameter, resulting in high ratings in the domain of “primary outcome” in all studies. The “eligibility,” that is, inclusion and exclusion criteria were also rated as rather pragmatic, as they were close to real-world conditions in almost all studies for the use of a health app; including mostly patients who would be eligible under real-world conditions as well. The organization was mostly rated (very) pragmatic as well, carrying out the study in the usual care environment.

Due to more extensive data collection, RCTs had a slightly lower average score in the domain of “follow-up,” mostly due to patient-reported outcomes. Non-RCTs, on the other hand, were rated as less pragmatic than RCTs in the domains of “primary analysis” and “recruitment” due to insufficient inclusion of all available data and sometimes extensive recruitment strategies. The highest rating of pragmatism was achieved when studies analyzed actual user data.

### RoB Assessment

**Figure 2:**
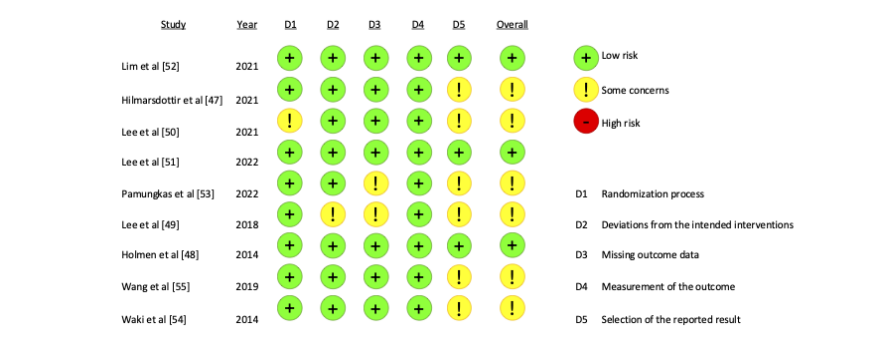
Cochrane risk of bias assessment of randomized controlled trials included in the meta-analysis [47-55]. D: domain.

Only 3 RCTs were rated as having a low RoB (Figure 2 [47-55]). All of the remaining RCTs were rated as having some concerns, with domain 5 raising concerns in all of them, due to missing study protocols. Missing blinding of participants was present in all studies and some studies also lacked blinding of the outcome assessor (domain 4). We nevertheless decided to rate the RoB as low in this respect, as the outcome of interest, the HbA_1c_, is a physiological parameter that is less likely to be affected by knowledge of the received intervention, when compared to patient-reported outcomes [26].

All of the non-RCTs had at least a serious (n=4), if not a critical (n=5) RoB according to ROBINS-I; the detailed ratings can be found in Multimedia Appendix 6. Due to missing information on clear time frames for assessment as well as patient flow, one study could not be ranked [57]. All of the ranked studies had a serious RoB due to confounding because no adjustment for possible confounders was performed or, as in the case of Berman et al [58], adjustment for postintervention variables was performed. The categories leading to critical risks were due to bias in the selection of participants. This particularly relates to observational studies that analyzed app users but excluded all participants who did not have a follow-up HbA_1c_, leading to exclusion rates of up to almost 80% [60] (Table 3). As it has to be assumed that reporting and continuation of the intervention is influenced by the intervention itself, the RoB of selection was judged to be critical. Another problem of RWD that led to a critical selection bias, was that start and follow-up times did not coincide for all users. Again, the lack of statistical adjustments to correct for missing data resulted in a selection bias and also did not offer evidence that the results were robust to missing data. Two of the studies also had a serious RoB in the category of measurement outcome due to self-reported HbA_1c_ values.

### Grading of Evidence

The grading of evidence was based on the meta-analyses results for the between group differences using 9 RCTs and the within-group differences using 12 RCTs as well as 11 observational studies. Using the GRADE Framework, the levels of certainty for the HbA_1c_ outcomes were rated as low quality of evidence for the RCTs and very low quality of evidence for the observational studies (Table 4).

**Table 4:** Summary of findings table.

Certainty assessment								Patients, n			Effect			Certainty
Studies, n	Study design	Risk of bias	Inconsistency	Indirectness	Imprecision	Other factors	App-based intervention		Standard care	Relative (95% CI)		Absolute (95% CI)		
9	RCTs^a^ (between groups)	Serious^b^	Not serious	Serious^c^	Not serious	None	523		522	—^d^		MD^e^ –0.36% (–0.59 to –0.14)	2/4 low	
10	Non-RCTs	Very serious^f^	Very serious^g^	Very serious^h^	Not serious	None	2358		271	—		MD –0.87% (–1.16 to –0.57)	1/4 very low	
9	RCTs (within-group)	Serious^b^	Not serious	Serious^c^	Not serious	None	523		522	—		MD –0.69% (–1.13 to –0.24)	2/4 low	

^a^RCT: randomized controlled trial.

^b^Mainly moderate risk of bias (RoB2-Tool).

^c^Mainly Asia, widely varying intervention duration.

^d^Not applicable.

^e^MD: mean difference.

^f^Serious to critical Risk Of Bias in Non-Randomized Studies of Interventions (ROBINS-I-Tool).

^g^High heterogeneity *I*^2^ 82%-94%.

^h^Mainly in the United States, widely varying intervention duration and intervention duration are often unclear.

For the between group differences in RCTs, this rating is due to a serious RoB, illustrated by the low to moderate RoB 2 rating. It also reflects a serious indirectness of the results, which arises from the RCTs being conducted primarily in Asia with widely varying intervention durations of 3 to 12 months. However, imprecision was not detected and neither was inconsistency, based on a low heterogeneity score of *I*^2^=19%. The same applies to publication bias, which was also not detected. For the RCTs used for the within-group differences, the same grading applies.

For the non-RCTs, the very low quality of evidence arose from a very serious RoB rating, pictured by the results of the ROBINS-I-Tool, which showed a serious to critical RoB in the non-RCTs (Multimedia Appendix 6). A high heterogeneity score of *I*^2^=82%-94% showed a high, very serious inconsistency. Due to studies being primarily conducted in the United States with intervention durations widely varying or remaining unclear, as is the nature of RWD, the quality was further downgraded for very serious indirectness. It was further downgraded for publication bias because funnel plots showed some asymmetry in favor of positive results. Imprecision however was not detected.

### Results of Meta-Analysis

#### Between Group Differences

**Figure 3:**
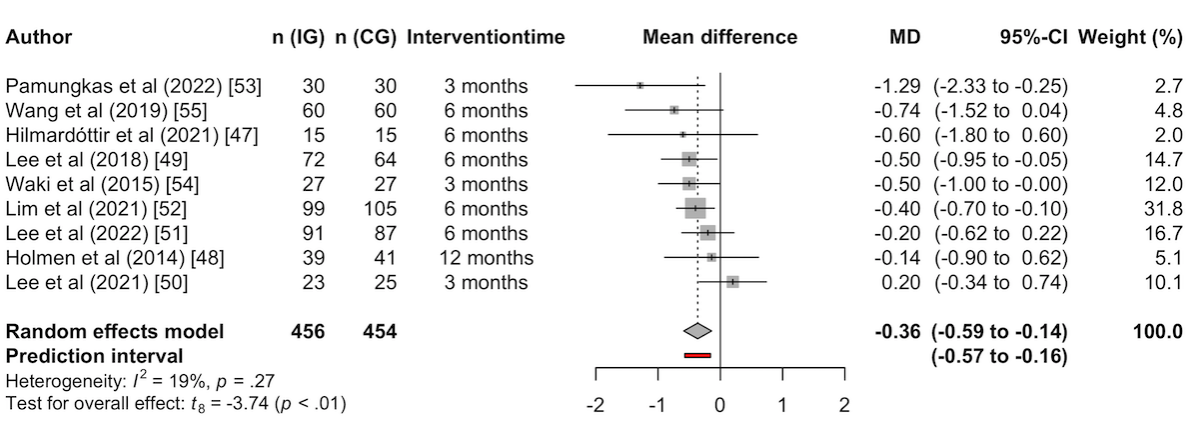
Meta-analysis of change in HbA1c after app-based lifestyle intervention meeting DiGA definition. DiGA: digitale Gesundheitsanwendung (digital health app); HbA1c: hemoglobin A1c (long-term blood glucose); MD: mean difference.

To assess the effect of app-based interventions compared to usual care, the results of the RCTs were pooled. The meta-analysis was performed independent of the intervention period because the number of studies that fell in the same category was too small. Most studies reported results after 6 months, two after 3 months, and one after 12 months. The average MD between IG and CG in the reduction of HbA_1c_ was –0.36% (95% CI –0.59% to –0.14%), favoring app-based interventions (Figure 3). The random effects model yielded significance implying that the group differences were significant (*P*<.001). The between-study heterogeneity can be assumed to be low with an *I*^2^ of 19% and the test for heterogeneity does not yield significance (*P*=.27). The prediction interval ranged from –0.57% to –0.16% and hence is very similar to the effect size, indicating robust results.

#### Within-Group Differences

**Figure 4:**
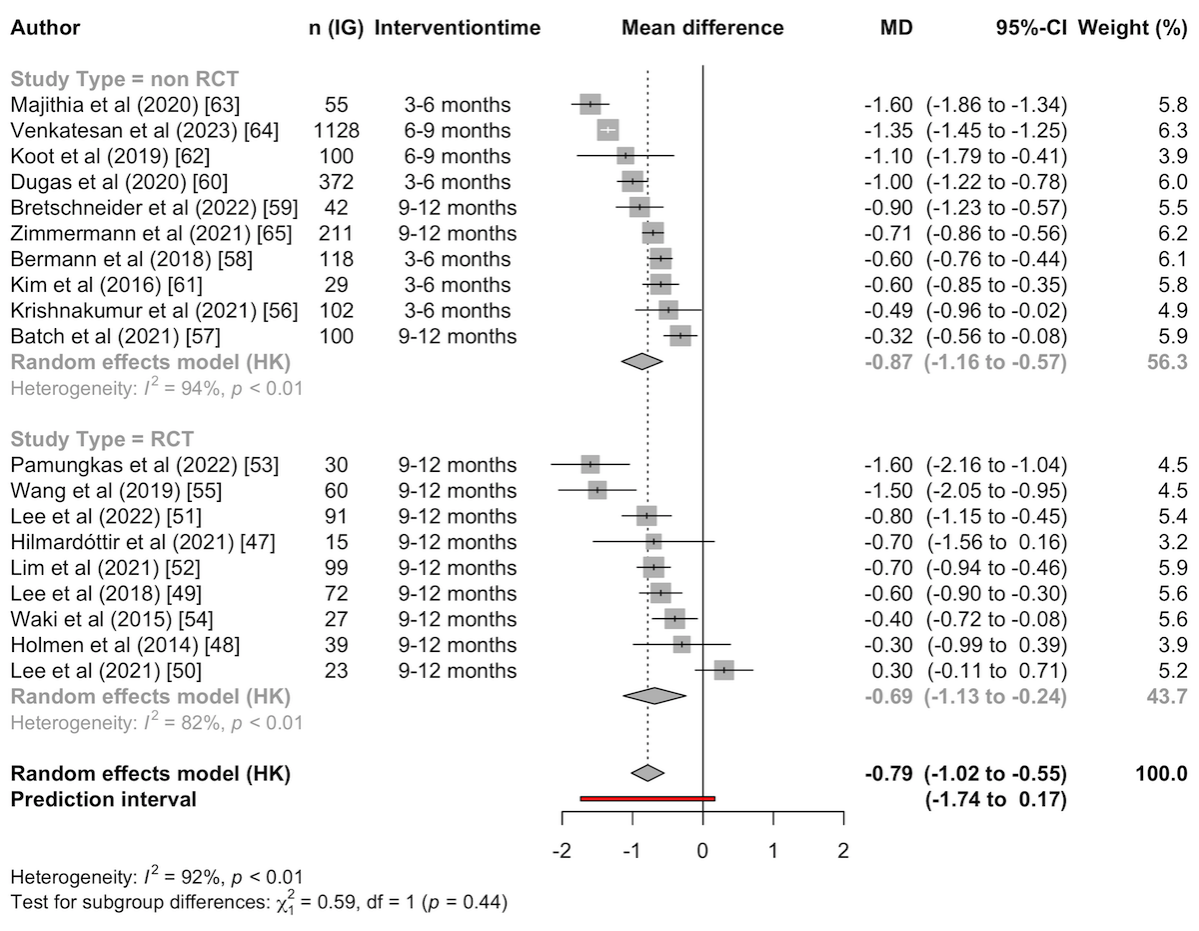
Meta-analysis of the pre-post effects within the IG of RCTs and non-RCTs. IG: intervention group; MD: mean difference; RCT: randomized controlled trial.

To compare the effects of RCTs and non-RCTs, pre-post effects within the IG of the apps were considered due to a lack of a CG in most non-RCTs. Again, the analysis was performed independent of the duration of the intervention. Non-RCTs had varying observation periods that, on average, were shorter compared to RCTs. The average mean pre-post reduction in HbA_1c_ levels is –0.79 (95% CI –1.02 to –0.55), not differing significantly (*P*=.44) between RCTs (–0.69, 95% CI –1.13 to –0.24) and non RCTs (–0.87, 95% CI –1.16 to –0.57; Figure 4). The high heterogeneity (*I*^2^=82%-94%) and the prediction interval that also included positive values indicate a potential lack of robustness of the results. The heterogeneity remains moderate to high (RCTs: 55%; non-RCTs: 59%) in the outlier analysis (Multimedia Appendix 7).

## Discussion

### Principal Findings

Overall, this systematic review and meta-analysis aimed at giving an overview of the current evidence regarding the efficacy and effectiveness of app-based interventions for the treatment of T2DM. For this, RCTs as well as non-RCTs were included. To our knowledge, this review is the first to include RCTs and non-RCTs for a pooled pre-post effect of digital lifestyle therapy on the HbA_1c_ in patients living with diabetes. Evidence from 9 RCTs shows that app-based interventions for the treatment of T2DM support patients in significantly reducing their HbA_1c_ levels by, on average, –0.36% and had favorable results compared to CGs receiving usual diabetes care. Looking at the within-group differences, patients were able to reduce their HbA_1c_ values on average by –0.79% after 3-12 months. While non-RCTs showed slightly higher reductions compared to the RCTs, the differences between study types were not significant. However, the heterogeneity (*I*^2^ statistic) for the within-group differences across study types, as well as within-study types was high, likely due to differences in the study duration and setting, methodology, and patients’ characteristics [66]. In contrast, the RCTs’ between-subject analysis resulted in a low heterogeneity. This shows the importance of controlling for baseline characteristics in statistical models to analyze outcomes. The pre-post effects only adjusted for confounding factors within their study population. In contrast, between group differences cancel out several effects unrelated to the intervention. First, the effect of the patient population characteristics itself. Second, the effect of the “usual care” that potentially differs between study sites (nationality, resources, study information provided to the participants, etc). Third, both groups are followed up over the same period, which allows controls for unexpected or spontaneous outcome-related events influencing the disease process of the whole population. As a result, only the between group differences show the isolated efficacy of the intervention, while within-group differences show the effectiveness of the intervention including external factors that might influence study outcomes, that is, the clinical effect under real-world conditions. This review shows that non-RCTs are likely to produce similar outcomes to RCTs per the effectiveness of interventions and as such can complement the evidence from RCTs. To address the limiting factor of a missing comparator in non-RCTs, appropriate artificial CGs could be used, such as matching methods or intraindividual cohorts. In fact, some of the included non-RCTs used the latter method.

### Comparison to Prior Work

The average additional mean HbA_1c_ reduction of –0.36% achieved with app-based interventions in comparison to usual care only, is comparable to previous systematic reviews on tele-medical treatment of diabetes that found mean group-differences of –0.44% [19] and –0.52% [21]. To reduce long-term diabetes-related complications, an HbA_1c_ reduction of 0.3% is considered clinically meaningful [67-69]. Stronger reductions in previous reviews could be due to broader inclusion criteria because the current review only included studies on apps that meet the DiGA definition and thus largely function through their technology independently of HCPs. Systematic reviews that analyze the efficacy of app-based interventions for type 1 diabetes mellitus and T2DM show that additional remote access to HCPs [19] as well as a higher frequency and intensity of feedback and interaction are associated with greater HbA_1c_ reductions [20]. Whether or not the feedback is automated or manual, on the other hand, might not be relevant [70].

### Pragmatism (PRECIS-2) Versus Quality of the Study (GRADE)

RCTs are designed such that the result is highly controlled, providing strong internal validity. However, this does not mean an RCT cannot be implemented in a real-world setting. The reasons for choosing a non-RCT design can be very diverse, with pragmatic implementation being a major aspect. In our study, the concept of pragmatism was used to judge the external validity of studies with the help of the PRECIS-2 tool. The most pragmatic studies were those that analyzed actual users of the app-based interventions [46,60]. Both RCTs and non-RCTs had, on average, high grades for pragmatism indicating (very) pragmatic trials and good external validity. Due to a lack of transparency and suitable statistical methods to account for missing data, non-RCTs had slightly lower grades than RCTs in the domains “primary analysis” and “recruitment.”

The GRADE ratings in both RCTs and non-RCTs further showed potential for improvement in future trials aiming for analyzing in-app user data or studies without a CG. First of all, improved statistical methods should be used to control for baseline covariates. Especially in studies analyzing user data or data collected from health records, clear information on data selection and suitable statistical methods are lacking. Particularly, in the absence of a CG, the handling of missing data needs to be clearly justified (eg, patients who do not use the app or do not provide any data). One way to improve the quality might be the use of preregistered analysis that predefines statistical methods and inclusion or exclusion criteria of patients [23]. Additional sensitivity analyses under different assumptions or conditions may increase the interpretability of results, as might blinding that so far is a major limitation of most digital interventions.

While non-RCTs in the context of explanatory trials show several limitations, they do have strengths in depicting results in a real-world context as pragmatic trials. However, the results of the PRECIS-2 rating showed that non-RCTs and RCTs included in our study can be classified as similarly pragmatic, and as such hold the potential to analyze interventions under real-world conditions to complement RCTs conducted in a controlled setting. Especially, in the context of fast-changing digital interventions, non-RCTs offer a cost- and time-efficient addition to RCTs. It is important to note that, for both RCTs and non-RCTs, the results of the study depend on the specific recruitment strategy and patient characteristics. The findings offer insights into the shortcomings of both RCTs and non-RCTs concerning pragmatism, however, they should be read with care, and translation of the evidence to other care settings should be made with great caution.

### Limitations

This study has several limitations. First, it cannot be ruled out that relevant publications were overseen in the course of the conducted search. However, this was counteracted by building extensive search terms, based on the PICO scheme, and by using text words as well as MeSH terms. In addition, the search was also conducted in three different databases, which might have further contributed to limiting the risk of selection bias. Second, the present results are limited to studies without long-term, follow-up periods, generating short-term rather than long-term evidence of the interventions. Other reviews suggest that the initialization of a treatment and as such the immediate short-term outcomes of treatment duration might be associated with better treatment success [21] making long-term and follow-up effects of particular interest. Third, even though the comparator of the included RCTs was theoretically the same, that is, usual care, in practice diabetes care differs between countries and could not be considered thoroughly which might have influenced the results. Due to the limited number of studies that fit the inclusion criteria, the heterogeneity between studies could not be accounted for in further subgroup analyses, for example, based on the time of the interventions. In fact, the results of the GRADE rating further resulted in (very) low evidence for the results of meta-analysis. Therefore, the results should be evaluated with caution. Finally, it is important to note that non-RCTs including real-world evidence exist on a broad continuum of possible settings. Unlike RCTs, which are more clearly defined per their study conditions, they vary greatly in their design and context. The PRECIS-2 tool was used to obtain an estimate of how pragmatic the delivery of digital care was in the different studies, including RCTs and non-RCTs. The PRECIS-2 tool was originally developed for designing pragmatic RCTs. As the authors leave freedom and flexibility in the usage of the tool, we applied it for the retrospective grading for RCTs as well as for non-RCTs. Downgrading of specific dimensions within the PRECIS tool depends on the deviation from the usual care condition, which can also vary across different settings. This makes it difficult to apply coherent criteria to a set of different studies.

### Conclusions

Overall, it can be concluded that app-based lifestyle interventions that meet the definition of DiGA can effectively reduce HbA_1c_ in patients with T2DM. This has been shown not only in RCTs but also in non-RCTs. While the latter still have several limitations per their design and statistical analyses, non-RCTs that implement suitable designs and methodologies have the potential to become an important source of complementary evidence, for example, in the context of postmarket analyses or piloting studies.

A DiGA approval requires high-quality evidence with minimal sources for biases, such as RCTs, before manufacturers can claim their medical product as a DiGA. Non-RCTs and in particular the analysis of in-app data can complement evidence from RCTs as a cost and time-efficient source of evidence to continuously monitor clinical outcomes of the medical device after being placed on the market.

Beyond the monitoring of the clinically relevant endpoints of DiGAs, in-app data can be a relevant addition to understanding patients’ needs and support postmarket analysis. As such the combination of evidence generated by both RCTs and non-RCTs is gaining relevance to develop the potential of DiGAs, for example, implemented in usual care as hybrid models. Moreover, introducing digital care solutions in the health care system may pave the way for artificial intelligence to further enhance the treatment of T2DM worldwide. Yet, future studies should aim for more methodological transparency and appropriate statistical evaluation procedures and methodologies to account for current limitations of non-RCTs, such as the missing comparator.

## Appendix

Multimedia Appendix 1 Search strings for systematic review.

Multimedia Appendix 2 Publication bias.

Multimedia Appendix 3 PRISMA checklist for systematic reviews. PRISMA: Preferred Reporting Items for Systematic Reviews and Meta-Analyses.

Multimedia Appendix 4 PRECIS-2 rating. PRECIS: Pragmatic Explanatory Continuum Indicator Summary.

Multimedia Appendix 5 Characteristics of included apps.

Multimedia Appendix 6 Risk of bias assessment for non-RCTs. RCT: randomized controlled trial.

Multimedia Appendix 7 Outlier analyses.

## Abbreviations

CG: control group
DiGA: digitale Gesundheitsanwendung (digital health app)
GRADE: Grading of Recommendations, Assessment, Development and Evaluation
HbA_1c_: hemoglobin A1c (long-term blood glucose)
HCP: health care professional
IG: intervention group
ITT: intention-to-treat
MD: mean difference
MeSH: Medical Subject Headings
PICO: Population; Intervention; Control; Outcome
PRECIS-2: Pragmatic Explanatory Continuum Indicator Summary
PRISMA: Preferred Reporting Items for Systematic Reviews and Meta-Analyses
RCT: randomized controlled trial
RoB: Risk of Bias
ROBINS-I: Risk Of Bias in Non-Randomized Studies of Interventions
RWD: real-world data
T2DM: type 2 diabetes mellitus

## Data Availability

Data sharing does not apply to this article as no data sets were generated or analyzed during this study.

## References

[1] (2020). Global Health Estimates 2019: deaths by cause, age, sex, by country and by region, 2000-2019.

[2] Sun H, Saeedi P, Karuranga S, Pinkepank M, Ogurtsova K, Duncan BB, Stein C, Basit A, Chan JCN, Mbanya JC, Pavkov ME, Ramachandaran A, Wild SH, James S, Herman WH, Zhang P, Bommer C, Kuo S, Boyko EJ, Magliano DJ (2022). IDF diabetes atlas: global, regional and country-level diabetes prevalence estimates for 2021 and projections for 2045. Diabetes Res Clin Pract.

[3] Saeedi P, Petersohn I, Salpea P, Malanda B, Karuranga S, Unwin N, Colagiuri S, Guariguata L, Motala AA, Ogurtsova K, Shaw JE, Bright D, Williams R, IDF Diabetes Atlas Committee (2019). Global and regional diabetes prevalence estimates for 2019 and projections for 2030 and 2045: results from the International Diabetes Federation Diabetes Atlas, 9 edition. Diabetes Res Clin Pract.

[4] Sarwar N, Gao P, Seshasai SRK, Gobin R, Kaptoge S, Di Angelantonio E, Ingelsson E, Lawlor DA, Selvin E, Stampfer M, Stehouwer CDA, Lewington S, Pennells L, Thompson A, Sattar N, White IR, Ray KK, Danesh J, Emerging Risk Factors Collaboration (2010). Diabetes mellitus, fasting blood glucose concentration, and risk of vascular disease: a collaborative meta-analysis of 102 prospective studies. Lancet.

[5] Schwarz PEH, Timpel P, Harst L, Greaves CJ, Ali MK, Lambert J, Weber MB, Almedawar MM, Morawietz H (2018). Blood sugar regulation for cardiovascular health promotion and disease prevention: JACC health promotion series. J Am Coll Cardiol.

[6] Teli M, Thato R, Rias YA (2023). Predicting factors of health-related quality of life among adults with type 2 diabetes: a systematic review. SAGE Open Nurs.

[7] Grant PJ, Aboyans V, Bailey C, Ceriello A, Delgado V, Cosentino (2020). 2019 ESC guidelines on diabetes, pre-diabetes, and cardiovascular diseases developed in collaboration with the EASD: the task force for diabetes, pre-diabetes, and cardiovascular diseases of the European Society of Cardiology (ESC) and the European Association for the Study of Diabetes (EASD). Rev Esp Cardiol Engl Ed.

[8] ElSayed NA, Aleppo G, Aroda VR, Bannuru RR, Brown FM, Bruemmer D, Collins BS, Hilliard ME, Isaacs D, Johnson EL, Kahan S, Khunti K, Leon J, Lyons SK, Perry ML, Prahalad P, Pratley RE, Seley JJ, Stanton RC, Gabbay RA, on behalf of the American Diabetes Association (2023). 2. Classification and diagnosis of diabetes: standards of care in diabetes-2023. Diabetes Care.

[9] (2016). Global report on diabetes.

[10] Arzneimittelkommission Der Deutschen Apotheker (AMK), Arzneimittelkommission Der Deutschen Ärzteschaft (AkdÄ), Deutsche Dermatologische Gesellschaft E. V. (DDG), Deutsche Diabetes Gesellschaft E. V. (DDG), Deutsche Gesellschaft Der Plastischen RUÄCEV (DGPRAEC), Deutsche Gesellschaft Für Allgemeinmedizin Und Familienmedizin E. V. (DEGAM), et al. NVL Typ-2-Diabetes ? Teilpublikation der Langfassung, 2. Auflage [Internet]. Bundesärztekammer (BÄK); Kassenärztliche Bundesvereinigung (KBV); Arbeitsgemeinschaft der Wissenschaftlichen Medizinischen Fachgesellschaften (AWMF).

[11] Landgraf R, Heinemann L, Schleicher E, Gerdes C, Petersmann A, Müller-Wieland D, Müller UA, Freckmann G, Thaler M, Ziegler AG, Kleinwechter H, Nauck M (2022). Definition, klassifikation, diagnostik und differenzialdiagnostik des diabetes mellitus: update 2022. Diabetol Stoffwechs.

[12] Aberle J, Lautenbach A, Meyhöfer S, Schmid SM, Selig L, Blüher M (2020). Adipositas und diabetes. Diabetol Stoffwechs.

[13] McKenzie AL, Hallberg SJ, Creighton BC, Volk BM, Link TM, Abner MK, Glon RM, McCarter JP, Volek JS, Phinney SD (2017). A novel intervention including individualized nutritional recommendations reduces hemoglobin A1c level, medication use, and weight in type 2 diabetes. JMIR Diabetes.

[14] National Institute for Health and Care Excellence (NICE) (2015). Type 2 diabetes in adults: management.

[15] Schram MT, Baan CA, Pouwer F (2009). Depression and quality of life in patients with diabetes: a systematic review from the European Depression In Diabetes (EDID) research consortium. Curr Diabetes Rev.

[16] ElSayed NA, Aleppo G, Aroda VR, Bannuru RR, Brown FM, Bruemmer D, Collins BS, Hilliard ME, Isaacs D, Johnson EL, Kahan S, Khunti K, Leon J, Lyons SK, Perry ML, Prahalad P, Pratley RE, Seley JJ, Stanton RC, Gabbay RA, on behalf of the American Diabetes Association (2022). 7. Diabetes technology: standards of care in diabetes-2023. Diabetes Care.

[17] Digitale-Versorgung-Gesetz. Bundesregierung.

[18] Kramer U (2023). DiGA-fast track – blueprint für digitalturbo in Europa?. HealthOn.

[19] Bonoto BC, de Araújo VE, Godói IP, de Lemos LLP, Godman B, Bennie M, Diniz LM, Junior AAG (2017). Efficacy of mobile apps to support the care of patients with diabetes mellitus: a systematic review and meta-analysis of randomized controlled trials. JMIR mHealth uHealth.

[20] Byambasuren O, Sanders S, Beller E, Glasziou P (2018). Prescribable mHealth apps identified from an overview of systematic reviews. npj Digit Med.

[21] Timpel P, Oswald S, Schwarz PEH, Harst L (2020). Mapping the evidence on the effectiveness of telemedicine interventions in diabetes, dyslipidemia, and hypertension: an umbrella review of systematic reviews and meta-analyses. J Med Internet Res.

[22] BfArM (2023). Das fast-track-verfahren für digitale gesundheitsanwendungen (DiGA) nach § 139e SGB V. Bundesinstitut für Arzneimittel und Medizinprodukte.

[23] Baumfeld Andre E, Reynolds R, Caubel P, Azoulay L, Dreyer NA (2020). Trial designs using real-world data: the changing landscape of the regulatory approval process. Pharmacoepidemiol Drug Saf.

[24] Wicherski J, Schneider K, Zinserling J, Heß S, Haenisch B, Broich K (2023). Real-world-Daten in der Arzneimittelregulation – aktuelle Entwicklungen und Ausblick. Präv Gesundheitsf.

[25] Cave A, Kurz X, Arlett P (2019). Real-world data for regulatory decision making: challenges and possible solutions for Europe. Clin Pharmacol Ther.

[26] Hemkens LG (2021). [Benefit assessment of digital health applications-challenges and opportunities]. Bundesgesundheitsblatt-Gesundheitsforschung-Gesundheitsschutz.

[27] Khosla S, Tepie MF, Nagy MJ, Kafatos G, Seewald M, Marchese S, Liwing J (2021). The alignment of real-world evidence and digital health: realising the opportunity. Ther Innov Regul Sci.

[28] Lange S, Lauterberg J (2022). Pragmatischere randomisierte Studien mit Fokus auf Registerbasierung. Präv Gesundheitsf.

[29] Page MJ, McKenzie JE, Bossuyt PM, Boutron I, Hoffmann TC, Mulrow CD, Shamseer L, Tetzlaff JM, Akl EA, Brennan SE, Chou R, Glanville J, Grimshaw JM, Hróbjartsson A, Lalu MM, Li T, Loder EW, Mayo-Wilson E, McDonald S, McGuinness LA, Stewart LA, Thomas J, Tricco AC, Welch VA, Whiting P, Moher D (2021). The PRISMA 2020 statement: an updated guideline for reporting systematic reviews. BMJ.

[30] Kohl C, McIntosh EJ, Unger S, Haddaway NR, Kecke S, Schiemann J, Wilhelm R (2018). Online tools supporting the conduct and reporting of systematic reviews and systematic maps: a case study on CADIMA and review of existing tools. Environ Evid.

[31] Loudon K, Treweek S, Sullivan F, Donnan P, Thorpe KE, Zwarenstein M (2015). The PRECIS-2 tool: designing trials that are fit for purpose. BMJ.

[32] Sterne JAC, Savović J, Page MJ, Elbers RG, Blencowe NS, Boutron I, Cates CJ, Cheng HY, Corbett MS, Eldridge SM, Emberson JR, Hernán MA, Hopewell S, Hróbjartsson A, Junqueira DR, Jüni P, Kirkham JJ, Lasserson T, Li T, McAleenan A, Reeves BC, Shepperd S, Shrier I, Stewart LA, Tilling K, White IR, Whiting PF, Higgins JPT (2019). RoB 2: a revised tool for assessing risk of bias in randomised trials. BMJ.

[33] Sterne JA, Hernán MA, Reeves BC, Savović J, Berkman ND, Viswanathan M, Henry D, Altman DG, Ansari MT, Boutron I, Carpenter JR, Chan AW, Churchill R, Deeks JJ, Hróbjartsson A, Kirkham J, Jüni P, Loke YK, Pigott TD, Ramsay CR, Regidor D, Rothstein HR, Sandhu L, Santaguida PL, Schünemann HJ, Shea B, Shrier I, Tugwell P, Turner L, Valentine JC, Waddington H, Waters E, Wells GA, Whiting PF, Higgins JP (2016). ROBINS-I: a tool for assessing risk of bias in non-randomised studies of interventions. BMJ.

[34] Guyatt G, Oxman AD, Akl EA, Kunz R, Vist G, Brozek J, Norris S, Falck-Ytter Y, Glasziou P, DeBeer H, Jaeschke R, Rind D, Meerpohl J, Dahm P, Schünemann HJ (2011). GRADE guidelines: 1. Introduction-GRADE evidence profiles and summary of findings tables. J Clin Epidemiol.

[35] Harrer M, Cuijpers P, Furukawa TA, Ebert DD (2022). Chapter 9 publication bias. Doing meta-analysis in R.

[36] Harrer M, Cuijpers P, Furukawa TA, Ebert DD (2022). Chapter 3 effect sizes. Doing meta-analysis with R: a hands-on guide.

[37] Balduzzi S, Rücker G, Schwarzer G (2019). How to perform a meta-analysis with R: a practical tutorial. Evid Based Ment Health.

[38] Harrer M, Cuijpers P, Furukawa TA, Ebert DD (2022). Chapter 4 pooling effect sizes. Doing meta-analysis with R: a hands-on guide.

[39] Higgins JPT, Thompson SG, Spiegelhalter DJ (2009). A re-evaluation of random-effects meta-analysis. J R Stat Soc Ser A Stat Soc.

[40] Higgins JPT, Tianjing L, Deeks JJ (2022). Chapter 6: choosing effect measures and computing estimates of effect. Cochrane Handbook for Systematic Reviews of Interventions.

[41] Schünemann H, Brożek J, Guyatt G, Oxman A (2013). Handbook for grading quality of evidence and strength of recommendations.

[42] Harrer M, Cuijpers P, Furukawa TA, Ebert DD (2022). Chapter 5 between-study heterogeneity. Doing meta-analysis with R: a hands-on guide.

[43] Lee MK, Lee DY, Ahn HY, Park CY (2021). A novel user utility score for diabetes management using tailored mobile coaching: secondary analysis of a randomized controlled trial. JMIR mHealth uHealth.

[44] Lim SL, Tay MHJ, Ong KW, Johal J, Yap QV, Chan YH, Yeo GKN, Khoo CM, Yaxley A (2022). Association between mobile health app engagement and weight loss and glycemic control in adults with type 2 diabetes and prediabetes (D'LITE Study): prospective cohort study. JMIR Diabetes.

[45] Torbjørnsen A, Jenum AK, Småstuen MC, Arsand E, Holmen H, Wahl AK, Ribu L (2014). A low-intensity mobile health intervention with and without health counseling for persons with type 2 diabetes, part 1: baseline and short-term results from a randomized controlled trial in the Norwegian part of RENEWING HEALTH. JMIR mHealth uHealth.

[46] Dixon RF, Zisser H, Layne JE, Barleen NA, Miller DP, Moloney DP, Majithia AR, Gabbay RA, Riff J (2020). A virtual type 2 diabetes clinic using continuous glucose monitoring and endocrinology visits. J Diabetes Sci Technol.

[47] Hilmarsdóttir E, Sigurðardóttir ÁK, Arnardóttir RH (2021). A digital lifestyle program in outpatient treatment of type 2 diabetes: a randomized controlled study. J Diabetes Sci Technol.

[48] Holmen H, Torbjørnsen A, Wahl AK, Jenum AK, Småstuen MC, Arsand E, Ribu L (2014). A mobile health intervention for self-management and lifestyle change for persons with type 2 diabetes, part 2: one-year results from the Norwegian randomized controlled trial RENEWING HEALTH. JMIR mHealth uHealth.

[49] Lee DY, Park J, Choi D, Ahn HY, Park SW, Park CY (2018). The effectiveness, reproducibility, and durability of tailored mobile coaching on diabetes management in policyholders: a randomized, controlled, open-label study. Sci Rep.

[50] Lee SE, Park SK, Park YS, Kim KA, Choi HS, Oh SW (2021). Effects of short-term mobile application use on weight reduction for patients with type 2 diabetes. J Obes Metab Syndr.

[51] Lee EY, Cha SA, Yun JS, Lim SY, Lee JH, Ahn YB, Yoon KH, Hyun MK, Ko SH (2022). Efficacy of personalized diabetes self-care using an electronic medical record-integrated mobile app in patients with type 2 diabetes: 6-month randomized controlled trial. J Med Internet Res.

[52] Lim SL, Ong KW, Johal J, Han CY, Yap QV, Chan YH, Chooi YC, Zhang ZP, Chandra CC, Thiagarajah AG, Khoo CM (2021). Effect of a smartphone app on weight change and metabolic outcomes in Asian adults with type 2 diabetes: a randomized clinical trial. JAMA Netw Open.

[53] Pamungkas RA, Usman AM, Chamroonsawasdi K, Abdurrasyid (2022). A smartphone application of diabetes coaching intervention to prevent the onset of complications and to improve diabetes self-management: a randomized control trial. Diabetes Metab Syndr.

[54] Waki K, Fujita H, Uchimura Y, Omae K, Aramaki E, Kato S, Lee H, Kobayashi H, Kadowaki T, Ohe K (2014). DialBetics: a novel smartphone-based self-management support system for type 2 diabetes patients. J Diabetes Sci Technol.

[55] Wang Y, Li M, Zhao X, Pan X, Lu M, Lu J, Hu Y (2019). Effects of continuous care for patients with type 2 diabetes using mobile health application: a randomised controlled trial. Int J Health Plann Manage.

[56] Krishnakumar A, Verma R, Chawla R, Sosale A, Saboo B, Joshi S, Shaikh M, Shah A, Kolwankar S, Mattoo V (2021). Evaluating glycemic control in patients of South Asian origin with type 2 diabetes using a digital therapeutic platform: analysis of real-world data. J Med Internet Res.

[57] Batch BC, Spratt SE, Blalock DV, Benditz C, Weiss A, Dolor RJ, Cho AH (2021). General behavioral engagement and changes in clinical and cognitive outcomes of patients with type 2 diabetes using the Time2Focus mobile app for diabetes education: pilot evaluation. J Med Internet Res.

[58] Berman MA, Guthrie NL, Edwards KL, Appelbaum KJ, Njike VY, Eisenberg DM, Katz DL (2018). Change in glycemic control with use of a digital therapeutic in adults with type 2 diabetes: cohort study. JMIR Diabetes.

[59] Bretschneider MP, Klásek J, Karbanová M, Timpel P, Herrmann S, Schwarz PEH (2022). Impact of a digital lifestyle intervention on diabetes self-management: a pilot study. Nutrients.

[60] Dugas M, Wang W, Crowley K, Iyer AK, Peeples M, Shomali M, Gao GG (2022). Engagement and outcomes associated with contextual annotation features of a digital health solution. J Diabetes Sci Technol.

[61] Kim EK, Kwak SH, Baek S, Lee SL, Jang HC, Park KS, Cho YM (2016). Feasibility of a patient-centered, smartphone-based, diabetes care system: a pilot study. Diabetes Metab J.

[62] Koot D, Goh PSC, Lim RSM, Tian Y, Yau TY, Tan NC, Finkelstein EA (2019). A mobile lifestyle management program (GlycoLeap) for people with type 2 diabetes: single-arm feasibility study. JMIR mHealth uHealth.

[63] Majithia AR, Kusiak CM, Armento Lee A, Colangelo FR, Romanelli RJ, Robertson S, Miller DP, Erani DM, Layne JE, Dixon RF, Zisser H (2020). Glycemic outcomes in adults with type 2 diabetes participating in a continuous glucose monitor-driven virtual diabetes clinic: prospective trial. J Med Internet Res.

[64] Venkatesan A, Zimmermann G, Rawlings K, Ryan C, Voelker L, Edwards C (2023). Improvements in glycemic control and depressive symptoms among adults with type 2 diabetes: retrospective study. JMIR Form Res.

[65] Zimmermann G, Venkatesan A, Rawlings K, Scahill MD (2021). Improved glycemic control with a digital health intervention in adults with type 2 diabetes: retrospective study. JMIR Diabetes.

[66] Cuijpers P, Weitz E, Cristea IA, Twisk J (2017). Pre-post effect sizes should be avoided in meta-analyses. Epidemiol Psychiatr Sci.

[67] Lind M, Odén A, Fahlén M, Eliasson B (2010). The shape of the metabolic memory of HbA_1c_: re-analysing the DCCT with respect to time-dependent effects. Diabetologia.

[68] Lind M, Odén A, Fahlén M, Eliasson B (2008). A systematic review of HbA_1c_ variables used in the study of diabetic complications. Diabetes Metab Syndr Clin Res Rev.

[69] Brinkworth GD, Wycherley TP, Taylor PJ, Thompson CH (2022). A health care professional delivered low carbohydrate diet program reduces body weight, haemoglobin A1c, diabetes medication use and cardiovascular risk markers-a single-arm intervention analysis. Nutrients.

[70] Shen Y, Wang F, Zhang X, Zhu X, Sun Q, Fisher E, Sun X (2018). Effectiveness of internet-based interventions on glycemic control in patients with type 2 diabetes: meta-analysis of randomized controlled trials. J Med Internet Res.

