# Supplementary material for: Effect of Multimodal App-Based Interventions on Glycemic Control in Patients With Type 2 Diabetes: Systematic Review and Meta-Analysis
Source: J Med Internet Res. 2025 Jan 24;27:e54324. doi: 10.2196/54324 (PMC11806272; doi:10.2196/54324)
Supplement: Multimedia Appendix 1 [file jmir_v27i1e54324_app1.docx]

**Table S1.** Search string PubMed.

| **PubMed** | | | |
| --- | --- | --- | --- |
| Date: 27.01.2023 | | | |
| Filter: 10 years, english, german | | | |
|  | **Query** | **Search Details** | **Results** |
| **1** | ("diabetes mellitus, type 2"[mh] OR ("diabet*"[tw] AND (type 2[tw] OR type two[tw]))) NOT (child*[tw] OR teen*[tw] OR adolesc*[tw] OR "juvenile"[tw] OR "pregna*"[tw] OR "gestation*"[tw] OR animal[tw]) | ("diabetes mellitus, type 2"[MeSH Terms] OR ("diabet*"[Text Word] AND ("type 2"[Text Word] OR "type two"[Text Word]))) NOT ("child*"[Text Word] OR "teen*"[Text Word] OR "adolesc*"[Text Word] OR "juvenile"[Text Word] OR "pregna*"[Text Word] OR "gestation*"[Text Word] OR "animal"[Text Word]) | 196,391 |
| **2** | "mobile applications"[mh] OR "app"[tw] OR appli*[tw] OR "smartphone"[tw] OR "tablet"[tw] OR "mobile device"[tw] OR "mHealth"[tw] OR "m-Health"[tw] OR "DTx"[tw] | "mobile applications"[MeSH Terms] OR "app"[Text Word] OR "appli*"[Text Word] OR "smartphone"[Text Word] OR "tablet"[Text Word] OR "mobile device"[Text Word] OR "mHealth"[Text Word] OR "m-Health"[Text Word] OR "DTx"[Text Word] | 2,679,980 |
| **3** | ("exercise therapy"[mh] OR "diet therapy"[mh] OR "training"[tw] OR "exercise"[tw] OR "physical activity"[tw] OR "nutrition"[tw] OR "diet"[tw] OR "lifestyle"[tw]) NOT "Drug Therapy"[mh] | ("exercise therapy"[MeSH Terms] OR "diet therapy"[MeSH Terms] OR "training"[Text Word] OR "exercise"[Text Word] OR "physical activity"[Text Word] OR "nutrition"[Text Word] OR "diet"[Text Word] OR "lifestyle"[Text Word]) NOT "Drug Therapy"[MeSH Terms] | 1,668,471 |

| **4** | ("glycated hemoglobin"[mh] OR "HbA1c"[tw] OR "A1c"[tw] OR glycated hemoglobin[tw]) OR (weight[tw] OR self management[tw] OR depress*[tw]) | "glycated hemoglobin"[MeSH Terms] OR "HbA1c"[Text Word] OR "A1c"[Text Word] OR "glycated hemoglobin"[Text Word] OR "weight"[Text Word] OR "self management"[Text Word] OR "depress*"[Text Word] | 1,887,524 |
| --- | --- | --- | --- |
| **5** | #1 AND #2 AND #3 AND #4 | (("diabetes mellitus, type 2"[MeSH Terms] OR ("diabet*"[Text Word] AND ("type 2"[Text Word] OR "type two"[Text Word]))) NOT ("child*"[Text Word] OR "teen*"[Text Word] OR "adolesc*"[Text Word] OR "juvenile"[Text Word] OR "pregna*"[Text Word] OR "gestation*"[Text Word] OR "animal"[Text Word])) AND ("mobile applications"[MeSH Terms] OR "app"[Text Word] OR "appli*"[Text Word] OR "smartphone"[Text Word] OR "tablet"[Text Word] OR "mobile device"[Text Word] OR "mHealth"[Text Word] OR "m-Health"[Text Word] OR "DTx"[Text Word]) AND (("exercise therapy"[MeSH Terms] OR "diet therapy"[MeSH Terms] OR "training"[Text Word] OR "exercise"[Text Word] OR "physical activity"[Text Word] OR "nutrition"[Text Word] OR "diet"[Text Word] OR "lifestyle"[Text Word]) NOT "Drug Therapy"[MeSH Terms]) AND ("glycated hemoglobin"[MeSH Terms] OR "HbA1c"[Text Word] OR "A1c"[Text Word] OR "glycated hemoglobin"[Text Word] OR ("weight"[Text Word] OR "self management"[Text Word] OR "depress*"[Text Word])) | 940 |
| **6** | #5 NOT study protocol | (((("diabetes mellitus, type 2"[MeSH Terms] OR ("diabet*"[Text Word] AND ("type 2"[Text Word] OR "type two"[Text Word]))) NOT ("child*"[Text Word] OR "teen*"[Text Word] OR "adolesc*"[Text Word] OR "juvenile"[Text Word] OR "pregna*"[Text Word] OR "gestation*"[Text Word] OR "animal"[Text Word])) AND ("mobile applications"[MeSH Terms] OR "app"[Text Word] OR "appli*"[Text Word] OR "smartphone"[Text Word] OR "tablet"[Text Word] OR "mobile device"[Text Word] OR "mHealth"[Text Word] OR "m-Health"[Text Word] OR "DTx"[Text Word]) AND (("exercise therapy"[MeSH Terms] OR "diet therapy"[MeSH Terms] OR "training"[Text Word] OR "exercise"[Text Word] OR "physical activity"[Text Word] OR "nutrition"[Text Word] OR "diet"[Text Word] OR "lifestyle"[Text Word]) NOT "Drug Therapy"[MeSH Terms]) AND ("glycated hemoglobin"[MeSH Terms] OR "HbA1c"[Text Word] OR "A1c"[Text Word] OR "glycated hemoglobin"[Text Word] OR ("weight"[Text Word] OR "self management"[Text Word] OR "depress*"[Text Word]))) NOT (("studies"[All Fields] OR "study"[All Fields] OR "study s"[All Fields] OR "studying"[All Fields] OR "studys"[All Fields]) AND ("protocol"[All Fields] OR "protocol s"[All Fields] OR "protocolized"[All Fields] OR "protocols"[All Fields]))) AND ((y_10[Filter]) AND (english[Filter] OR german[Filter])) | **630** |

**Table S2.** Search string LIVIVO.

| **LIVIVO** | | |
| --- | --- | --- |
| Date: 27.01.2023 | | |
| Filter: 10years, english, german, article, MEDLINE excluded | |  |
|  | **Query** | **Results** |
| **1** | (type 2 diabetes) AND (HbA1c OR glycated hemoglobin OR weight OR self management OR depression) AND (app OR smartphone OR tablet OR mobile device OR mHealth OR DTx) AND (training OR exercise OR nutrition OR diet OR lifestyle) | **109** |

**Table S3.** Search string Cochrane.

| **Cochrane** | | |
| --- | --- | --- |
| Date: 31.01.2023 | | |
| Filter: 10 years, english, german | | |
| **ID** | **Search** | **Results** |
| #1 | MeSH descriptor: [Diabetes Mellitus, Type 2] explode all trees | 20469 |
| #2 | (("diabet*" NEXT ("type 2" OR "type two"))) (Word variations have been searched) | 754 |
| #3 | (("child*" OR "teen*" OR "adolesc*" OR "juvenile" OR "pregna*" OR "gestation*" OR animal)) (Word variations have been searched) | 341437 |
| #4 | MeSH descriptor: [Glycated Hemoglobin A] explode all trees | 6469 |
| #5 | (("HbA1c" OR "A1c" OR "glycated hemoglobin" OR "weight" OR "self management" OR "depress*")) (Word variations have been searched) | 269084 |
| #6 | (("HbA1c" OR "A1c" OR "glycated hemoglobin" OR "weight" OR "self management" OR "German Diabetes Risk Score" OR "wellbeing")) (Word variations have been searched) | 193687 |
| #7 | MeSH descriptor: [Mobile Applications] explode all trees | 1165 |
| #8 | ("app" OR **"applic*"** OR "smartphone" OR "tablet" OR "mobile device" OR "mHealth" OR "m-Health" OR "DTx") | 53623 |
| #9 | MeSH descriptor: [Exercise Therapy] explode all trees | 16556 |
| #10 | MeSH descriptor: [Diet Therapy] explode all trees | 6696 |
| #11 | MeSH descriptor: [Risk Reduction Behavior] explode all trees | 1919 |
| #12 | MeSH descriptor: [Life Style] explode all trees | 6403 |
| #13 | (training OR "exercise" OR "physical activity" OR "nutrition" OR "diet" OR "lifestyle") (Word variations have been searched) | 338582 |
| #14 | MeSH descriptor: [Drug Therapy] explode all trees | 148743 |
| #15 | #4 OR #6 | 181716 |
| #16 | #7 OR #8 | 53810 |
| #17 | #9 OR #10 OR #11 OR #12 OR #13 | 308281 |
| #18 | (#1 OR #2) NOT #3 | 8723 |
| #19 | #4 OR #5 | 163276 |
| #20 | #7 OR #8 | 53810 |
| #21 | #9 OR #10 OR #11 OR #12 OR #13 | 308281 |
| #22 | (#18 AND #19 AND #20 AND #21) NOT #14 | 54 |
