# Supplementary material for: Effect of Multimodal App-Based Interventions on Glycemic Control in Patients With Type 2 Diabetes: Systematic Review and Meta-Analysis
Source: J Med Internet Res. 2025 Jan 24;27:e54324. doi: 10.2196/54324 (PMC11806272; doi:10.2196/54324)
Supplement: Multimedia Appendix 2 [file jmir_v27i1e54324_app2.pdf]

To assess a potential publication bias, funnel plots are used (Figures 5 and 6). The funnel plot for between-group differences based on RCTs only appears asymmetrical because of a lack of studies with high SE and low effect (MD). Compared to this plot, for the within-group differences from RCTs combined with non-RCTs, the funnel is shifted more strongly to the left, due to the higher effect. In addition, the distribution of studies seems more symmetrical compared to the funnel plot for RCTs only which suggests that no or little publication bias can be assumed.

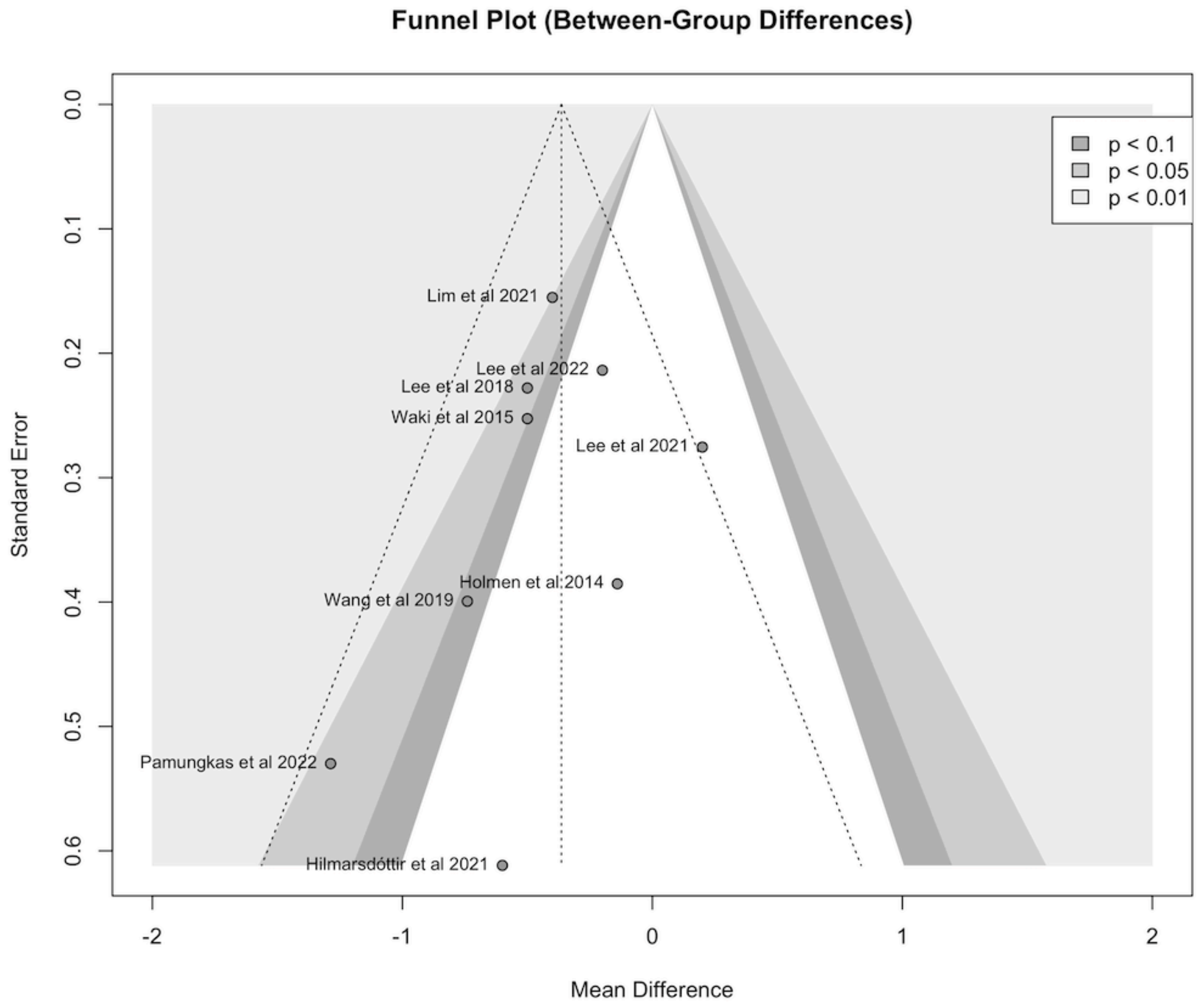

This is a Multimedia Appendix to a full manuscript published in the J Med Internet Res. For full copyright and citation information see http://dx.doi.org/10.2196/jmir.10.2196/54324

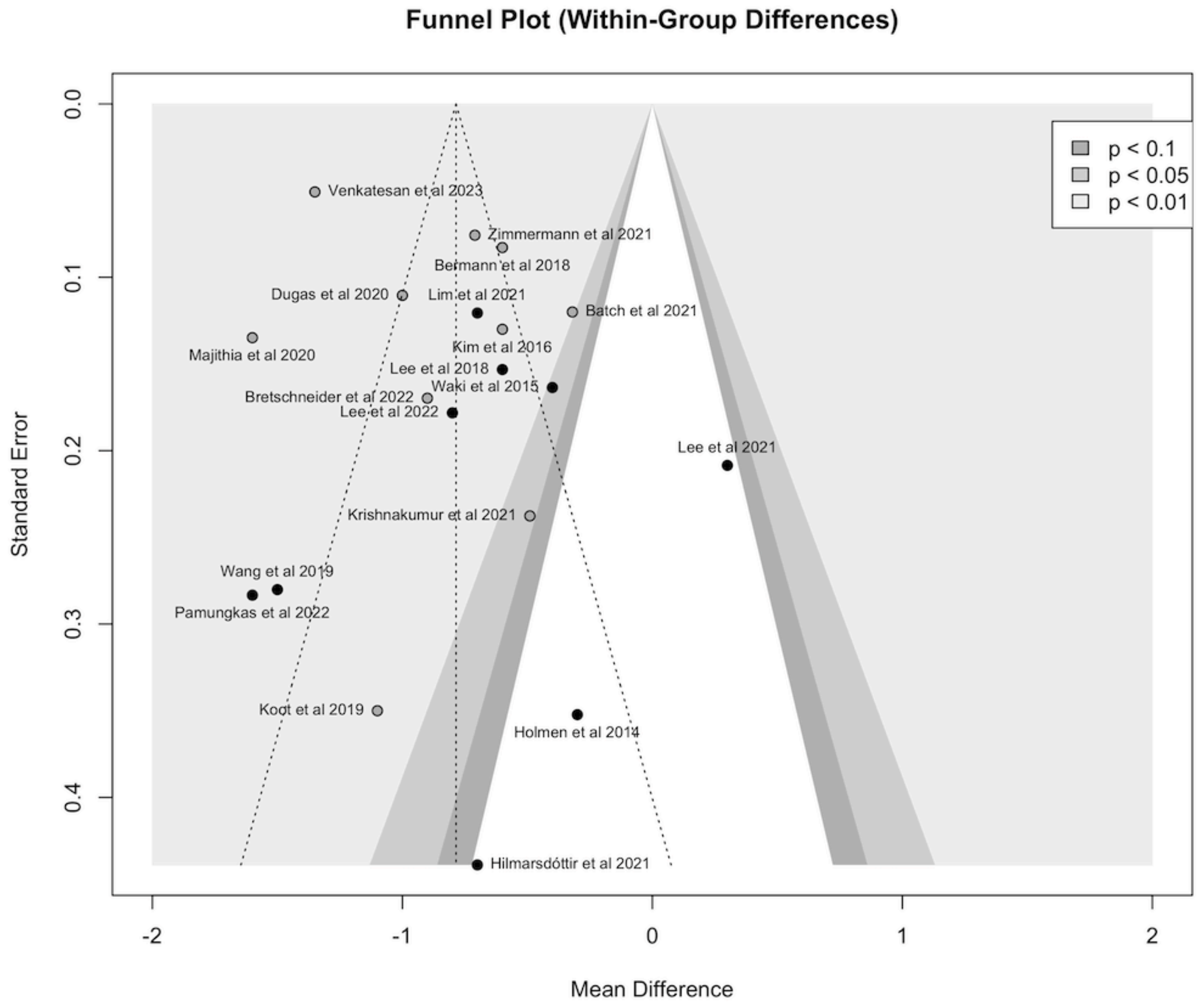

This is a Multimedia Appendix to a full manuscript published in the J Med Internet Res. For full copyright and citation information see http://dx.doi.org/10.2196/jmir.10.2196/54324

Literature

1. Lim SL, Ong KW, Johal J, Han CY, Yap QV, Chan YH, et al. Effect of a Smartphone App on Weight Change and Metabolic Outcomes in Asian Adults With Type 2 Diabetes: A Randomized Clinical Trial. JAMA Netw Open; 2021;4(6):e2112417. DOI: 10.1001/jamanetworkopen.2021.12417

2. Hilmarsdóttir E, Sigurðardóttir ÁK, Arnardóttir RH. A Digital Lifestyle Program in Outpatient Treatment of Type 2 Diabetes: A Randomized Controlled Study. J Diabetes Sci Technol; 2021;15(5):1134–41. DOI: 10.1177/1932296820942286

3. Lee SE, Park SK, Park YS, Kim KA, Choi HS, Oh SW. Effects of Short-term Mobile Application Use on Weight Reduction for Patients with Type 2 Diabetes. J Obes Metab Syndr; 2021;30(4):345–53. DOI: 10.7570/jomes21047

4. Lee EY, Cha SA, Yun JS, Lim SY, Lee JH, Ahn YB, et al. Efficacy of Personalized Diabetes Self-care Using an Electronic Medical Record-Integrated Mobile App in Patients With Type 2 Diabetes: 6-Month Randomized Controlled Trial. J Med Internet Res; 2022;24(7):e37430. DOI: 10.2196/37430

5. Pamungkas RA, Usman AM, Chamroonsawasdi K, Abdurrasyid. A smartphone application of diabetes coaching intervention to prevent the onset of complications and to improve diabetes self-management: A randomized control trial. Diabetes Metab Syndr; 2022;16(7):102537. DOI: 10.1016/j.dsx.2022.102537

6. Lee DY, Park J, Choi D, Ahn HY, Park SW, Park CY. The effectiveness, reproducibility, and durability of tailored mobile coaching on diabetes management in policyholders: A randomized, controlled, open-label study. Sci Rep; 2018;8(1):3642. DOI: 10.1038/s41598-018-22034-0

7. Holmen H, Torbjørnsen A, Wahl AK, Jenum AK, Småstuen MC, Arsand E, et al. A Mobile Health Intervention for Self-Management and Lifestyle Change for Persons With Type 2 Diabetes, Part 2: One-Year Results From the Norwegian Randomized Controlled Trial RENEWING HEALTH. JMIR Mhealth Uhealth; 2014;2(4):e57. DOI: 10.2196/mhealth.3882

8. Wang Y, Li M, Zhao X, Pan X, Lu M, Lu J, et al. Effects of continuous care for patients with type 2 diabetes using mobile health application: A randomised controlled trial. Int J Health Plann Mgmt; 2019 Jul;34(3):1025–35. DOI: 10.1002/hpm.2872

9. Waki K, Fujita H, Uchimura Y, Omae K, Aramaki E, Kato S, et al. DialBetics: A Novel Smartphone-based Self-management Support System for Type 2 Diabetes Patients. J Diabetes Sci Technol; 2014 Mar;8(2):209–15. DOI: 10.1177/1932296814526495

10. Bretschneider MP, Klásek J, Karbanová M, Timpel P, Herrmann S, Schwarz PEH. Impact of a Digital Lifestyle Intervention on Diabetes Self-Management: A Pilot Study. Nutrients; 2022;14(9):1810. DOI: 10.3390/nu14091810

11. Dugas M, Wang W, Crowley K, Iyer AK, Peeples M, Shomali M, et al. Engagement and Outcomes Associated with Contextual Annotation Features of a Digital Health Solution. J Diabetes Sci Technol; 2022;16(4):804–11. DOI: 10.1177/1932296820976409

12. Majithia AR, Kusiak CM, Armento Lee A, Colangelo FR, Romanelli RJ, Robertson S, et al. Glycemic Outcomes in Adults With Type 2 Diabetes Participating in a Continuous Glucose Monitor–Driven Virtual Diabetes Clinic: Prospective Trial. J Med Internet Res; 2020 Aug 28;22(8):e21778. DOI: 10.2196/21778

This is a Multimedia Appendix to a full manuscript published in the J Med Internet Res. For full copyright and citation information see http://dx.doi.org/10.2196/jmir.10.2196/54324

13. Koot D, Goh PSC, Lim RSM, Tian Y, Yau TY, Tan NC, et al. A Mobile Lifestyle Management Program (GlycoLeap) for People With Type 2 Diabetes: Single-Arm Feasibility Study. JMIR Mhealth Uhealth; 2019;7(5):e12965. DOI: 10.2196/12965

14. Venkatesan A, Zimmermann G, Rawlings K, Ryan C, Voelker L, Edwards C. Improvements in Glycemic Control and Depressive Symptoms Among Adults With Type 2 Diabetes: Retrospective Study. JMIR Form Res; 2023 Jan 13;0:e41880. DOI: 10.2196/41880

15. Zimmermann G, Venkatesan A, Rawlings K, Scahill MD. Improved Glycemic Control With a Digital Health Intervention in Adults With Type 2 Diabetes: Retrospective Study. JMIR Diabetes; 2021;6(2):e28033. DOI: 10.2196/28033

16. Kim EK, Kwak SH, Baek S, Lee SL, Jang HC, Park KS, et al. Feasibility of a Patient-Centered, Smartphone-Based, Diabetes Care System: A Pilot Study. Diabetes Metab J; 2016;40(3):192–201. DOI: 10.4093/dmj.2016.40.3.192

17. Berman MA, Guthrie NL, Edwards KL, Appelbaum KJ, Njike VY, Eisenberg DM, et al. Change in Glycemic Control With Use of a Digital Therapeutic in Adults With Type 2 Diabetes: Cohort Study. JMIR Diabetes; 2018;3(1):e4. DOI: 10.2196/diabetes.9591

18. Batch BC, Spratt SE, Blalock DV, Benditz C, Weiss A, Dolor RJ, et al. General Behavioral Engagement and Changes in Clinical and Cognitive Outcomes of Patients with Type 2 Diabetes Using the Time2Focus Mobile App for Diabetes Education: Pilot Evaluation. J Med Internet Res; 2021;23(1):e17537. DOI: 10.2196/17537

19. Krishnakumar A, Verma R, Chawla R, Sosale A, Saboo B, Joshi S, et al. Evaluating Glycemic Control in Patients of South Asian Origin With Type 2 Diabetes Using a Digital Therapeutic Platform: Analysis of Real-World Data. J Med Internet Res; 2021;23(3):e17908. DOI: 10.2196/17908

This is a Multimedia Appendix to a full manuscript published in the J Med Internet Res. For full copyright and citation information see http://dx.doi.org/10.2196/jmir.10.2196/54324
