# Supplementary material for: Effect of Multimodal App-Based Interventions on Glycemic Control in Patients With Type 2 Diabetes: Systematic Review and Meta-Analysis
Source: J Med Internet Res. 2025 Jan 24;27:e54324. doi: 10.2196/54324 (PMC11806272; doi:10.2196/54324)
Supplement: Multimedia Appendix 4 [file jmir_v27i1e54324_app4.docx]

## **PRECIS-2 Domains**

| **Eligibility (E):** | To what extent are the participants in the trial similar to those who would receive this intervention if it was part of usual care? |
| --- | --- |
| **Recruitment (R):** | How much extra effort is made to recruit participants over and above what that would be used in the usual care setting to engage with patients? |
| **Setting (S):** | How different is the setting of the trial and the usual care setting? |
| **Organization (O):** | How different are the resources, provider expertise and the organization of care delivery in the intervention arm of the trial and those available in usual care? |
| **Flexibility (delivery) (Fd):** | How different is the flexibility in how the intervention is delivered and the flexibility anticipated in usual care? |
| **Flexibility (adherence) (Fa):** | How different is the flexibility in how participants are monitored and encouraged to adhere to the intervention from the flexibility anticipated in usual care? |
| **Follow-Up (FU):** | How different is the intensity of measurement and follow-up of participants in the trial from the typical follow-up in usual care? |
| **Primary Outcome (PO):** | To what extent is the trial’s primary outcome directly relevant to participants? |
| **Primary Analysis (PA):** | To what extent are all data included in the analysis of the primary outcome? |

## **PRECIS-2 - Rating of RCTs**

Table S1 Represents the rating of pragmatism for RCTs, i.e., the rating for each domain as well as average rating per study and over all studies. If no explanation is given, studies are considered very pragmatic, i.e., almost equal to usual care. Explanations are given why RCTs deviate from usual care and are less pragmatic and more explanatory; answering the questions for each domain (see [PRECIS-2 Domains](#_heading=h.30j0zll)).

**Table S1.** Pragmatism rating and according explanation of RCTs.

|  | **E** | **R** | **S** | **O** | **Fd** | **Fa** | **FU** | **PO** | **PA** | **Mean** |
| --- | --- | --- | --- | --- | --- | --- | --- | --- | --- | --- |
| **Batch et al. 2021** | | | | | | | | | | |
| Rating | 5 | 4 | 4 | 5 | 4 | 4 | 4 | 5 | 4 | 4.3 |
| Expl. |  | one clinic only | specialized clinic |  | app-  introduction in person at study site | texts from first author | additional data (PROMs) |  | exclusion of lost to follow-up |  |
| **E.Y. Lee et al. 2022** | | | | | | | | | | |
| Rating | 3 | 4 | 4 | 4 | 4 | 5 | 2 | 5 | 5 | 4 |
| Expl. | self-management training necessary to participate;  special exclusion criteria | only two university-  affiliated hospitals | university-  Affiliated  hospitals | additional education  IG with HCP support: **3** | partly specific control |  | additional mobile app;  additional specialized data collection (PROMs, health events, bioimpedance analysis) |  |  |  |
| **Holmen et al. 2014** | | | | | | | | | | |
| Rating | 5 | 4 | 5 | 4 | 5 | 5 | 3 | 5 | 4 | 4.4 |
| Expl. |  | advertised |  | additional training of participants;  IG with HCP support: special nurse with special / extra support: **2** | with HCP support: additional very structured health counseling: **3** |  | additional data collection;  questionnaires via post if follow-up appointments in person was not possible |  | exclusion of lost to follow-up |  |
| **S. E. Lee et al. 2021** | | | | | | | | | | |
| Rating | 5 | 4 | 4 | 5 | 3 | 5 | 4 | 5 | 5 | 4.4 |
| Expl. |  | one clinic only | specialized outpatient clinic |  | two apps in combination (clear protocol);  not usual care context |  | additional and extensive data collection |  | exclusion of lost to follow-up |  |
| **Pamungkas et al. 2022** | | | | | | | | | | |
| Rating | 4 | 4 | 4 | 5 | 5 | 3 | 4 | 5 | 5 | 4.3 |
| Expl. | specific criteria | extra effort to select patients from additional centers | specific health centers |  |  | Encouragement;  online consultation;  phone calls | additional data (PROMs) |  |  |  |
| **Wang et al. 2019** | | | | | | | | | | |
| Rating | 4 | 4 | 4 | 5 | 5 | 5 | 5 | 5 | 5 | 4.6 |
| Expl. | narrow age group included | only one clinic | specific health unit |  |  |  |  |  |  |  |
| [2,3] | | | | | | | | | | |
| Rating | 4 | 4 | 4 | 3 | 3 | 4 | 2 | 5 | 4 | 3.6 |
| Expl. | specific criteria | only recruited from one insurance provider | hospital | extensive introduction in the beginning;  additional resources | complex and specific protocol | extra phone calls (organization) | home visits for data collection;  additional data collections (PROMs) |  | exclusion of lost to follow-up |  |
| **Waki et al. 2015** | | | | | | | | | | |
| Rating | 5 | 3 | 4 | 2 | 5 | 5 | 4 | 5 | 5 | 4.2 |
| Expl. |  | only one clinic;  advertised (posters);  test phase for app use | university hospital | additional staff;  Ressources;  extensive training of participants |  |  | additional data (PROMs) |  |  |  |
| **Lim et al. 2021 / 2022** | | | | | | | | | | |
| Rating | 5 | 4 | 5 | 3 | 3 | 2 | 2 | 5 | 5 | 3.7 |
| Expl. |  | specific health centers |  | extra study staff;  extensive introduction;  additional resources for participants | strict and specific protocol | regular and extensive interaction with participants | additional and extensive data collection (food diary, survey);  research staff measured (not usual care) |  |  |  |
| **Mean** | | | | | | | | | | |
|  | 4.4 | 3.8 | 4.2 | 4.0 | 4.1 | 4.2 | 3.3 | 5 | 4.6 | 4.2 |

Note: Expl., Explanation

## **PRECIS-2 - Rating of non-RCTs**

Table S2 Represents the rating of pragmatism for non-RCTs, i.e., the rating for each domain as well as average rating per study and over all studies. If no explanation is given, studies are considered very pragmatic, i.e., almost equal to usual care. Explanations are given why RCTs deviate from usual care and are less pragmatic and more explanatory; answering the questions for each domain (see [PRECIS-2 Domains](#_heading=h.30j0zll)).

**Table S2.** Pragmatism rating and according explanation of non-RCTs.

|  | **E** | **R** | **S** | **O** | **Fd** | **Fa** | **FU** | **PO** | **PA** | **Mean** |
| --- | --- | --- | --- | --- | --- | --- | --- | --- | --- | --- |
| **Batch et al. 2021** | | | | | | | | | | |
| Rating | 4 | 2 | 5 | 4 | 5 | 5 | 4 | 5 | 5 | 4.3 |
| Expl. | narrow inclusion criteria for HbA1c | investigator actively searched and contacted participants;  incentive |  | partly extra study specific effort (instructions) |  |  | extra data collection (survey;  CRF) |  |  |  |
| **Bermann et al. 2018** | | | | | | | | | | |
| Rating | 5 | 3 | 5 | 2 | 5 | 5 | 4 | 5 | 5 | 4.3 |
| Expl. |  | advertised;  incentive |  | extra study specific effort (phone calls; extra training study stuff; extensive additional support) |  |  | extra e-mail reminders to provide FU data by study staff |  |  |  |
| **Bretschneider et al. 2022** | | | | | | | | | | |
| Rating | 3 | 3 | 5 | 4 | 5 | 5 | 4 | 5 | 5 | 4.3 |
| Expl. | very specific inclusion criteria | Campaign;  extensive inclusion process;  incentives |  | extra study specific on-boarding |  |  | extra data collection (survey;  CRF) |  |  |  |
| **Dixon et al. 2020** | | | | | | | | | | |
| Rating | 5 | 5 | 5 | 5 | 5 | 5 | 5 | 5 | 4 | 4.8 |
| Expl. |  |  |  |  |  |  |  |  | exclusion of patients with missing data |  |
| **Dugas et al. 2020** | | | | | | | | | | |
| Rating | 5 | 5 | 5 | 5 | 5 | 5 | 5 | 5 | 4 | 4.8 |
| Expl. |  |  |  |  |  |  |  |  | exclusion of patients with missing data |  |
| **Kim et al. 2016** | | | | | | | | | | |
| Rating | 5 | 4 | 3 | 4 | 4 | 5 | 4 | 5 | 5 | 4.3 |
| Expl. |  | recruitment from only one clinic | university clinic;  face-to-face instructions | study specific website for study data | not allowed to change medication |  | extra data collection (survey;  CRF) |  |  |  |
| **Krishnakumur et al. 2021** | | | | | | | | | | |
| Rating | 4 | 3 | 5 | 4 | 5 | 5 | 4 | 5 | 4 | 4.3 |
| Expl. | partly specific criteria | campaign;  extensive inclusion process |  | extra Glucometer |  |  | study specific measures |  | exclusion of lost to follow-up |  |
| **Koot et al. 2019** | | | | | | | | | | |
| Rating | 4 | 2 | 4 | 2 | 5 | 5 | 5 | 5 | 5 | 4.1 |
| Expl. | specific criteria | investigator actively searched and contacted participant;  one clinic only;  incentives | community facility delivery | extra ressources for patients;  need to switch glucometer;  printed extra material |  |  |  |  |  |  |
| **Majithia et al. 2020** | | | | | | | | | | |
| Rating | 4 | 4 | 5 | 5 | 3 | 5 | 5 | 5 | 4 | 4.4 |
| Expl. | narrow HbA1c | only recruited from two clinics |  |  | Phone calls; extra sensor;  specific protocol for sensor use |  |  |  | non compliant patients excluded  (n = 3) |  |
| **Venkatesan et al. 2023** | | | | | | | | | | |
| Rating | 4 | 3 | 5 | 5 | 5 | 5 | 5 | 5 | 3 | 4.4 |
| Expl. | high risk population | campaign;  email announcement;  brochure;  one single payer client |  |  |  |  |  |  | high drop out (>50%) that was not part of analysis |  |
| **Zimmermann et al. 2021** | | | | | | | | | | |
| Rating | 5 | 4 | 5 | 5 | 5 | 5 | 5 | 5 | 3 | 4.6 |
| Expl. |  | campaign |  |  |  |  |  |  | high drop out (>50%) that was not part of analysis |  |
| **Mean** | | | | | | | | | | |
|  | 4.4 | 3.5 | 4.7 | 4.1 | 4.7 | 5 | 4.5 | 5 | 4.3 | 4.5 |

Note: Expl., Explanation

**Literature**

[1. Lim SL, Ong KW, Johal J, Han CY, Yap QV, Chan YH, et al. Effect of a Smartphone App on Weight Change and Metabolic Outcomes in Asian Adults With Type 2 Diabetes: A Randomized Clinical Trial. JAMA Netw Open; 2021;4(6):e2112417. DOI: 10.1001/jamanetworkopen.2021.12417](https://www.zotero.org/google-docs/?broken=A9nGv2)

[2. Lim SL, Tay MHJ, Ong KW, Johal J, Yap QV, Chan YH, et al. Association Between Mobile Health App Engagement and Weight Loss and Glycemic Control in Adults With Type 2 Diabetes and Prediabetes (D’LITE Study):  Prospective Cohort Study. JMIR Diabetes; 2022;7(3):e35039. DOI: 10.2196/35039](https://www.zotero.org/google-docs/?broken=rrXgnf)

[3. Hilmarsdóttir E, Sigurðardóttir ÁK, Arnardóttir RH. A Digital Lifestyle Program in Outpatient Treatment of Type 2 Diabetes: A Randomized Controlled Study. J Diabetes Sci Technol; 2021;15(5):1134–41. DOI: 10.1177/1932296820942286](https://www.zotero.org/google-docs/?broken=Db0Kdy)

[4. Lee SE, Park SK, Park YS, Kim KA, Choi HS, Oh SW. Effects of Short-term Mobile Application Use on Weight Reduction for Patients with Type 2 Diabetes. J Obes Metab Syndr; 2021;30(4):345–53. DOI: 10.7570/jomes21047](https://www.zotero.org/google-docs/?broken=md8rJS)

[5. Lee EY, Cha SA, Yun JS, Lim SY, Lee JH, Ahn YB, et al. Efficacy of Personalized Diabetes Self-care Using an Electronic Medical Record-Integrated Mobile App in Patients With Type 2 Diabetes: 6-Month Randomized  Controlled Trial. J Med Internet Res; 2022;24(7):e37430. DOI: 10.2196/37430](https://www.zotero.org/google-docs/?broken=F6ZCDC)

[6. Pamungkas RA, Usman AM, Chamroonsawasdi K, Abdurrasyid. A smartphone application of diabetes coaching intervention to prevent the onset of complications and to improve diabetes self-management: A randomized control  trial. Diabetes Metab Syndr; 2022;16(7):102537. DOI: 10.1016/j.dsx.2022.102537](https://www.zotero.org/google-docs/?broken=I9tb7d)

[7. Lee DY, Park J, Choi D, Ahn HY, Park SW, Park CY. The effectiveness, reproducibility, and durability of tailored mobile coaching on diabetes management in policyholders: A randomized, controlled, open-label study. Sci Rep; 2018;8(1):3642. DOI: 10.1038/s41598-018-22034-0](https://www.zotero.org/google-docs/?broken=EZi92K)

[8. Lee MK, Lee DY, Ahn HY, Park CY. A Novel User Utility Score for Diabetes Management Using Tailored Mobile Coaching: Secondary Analysis of a Randomized Controlled Trial. JMIR Mhealth Uhealth; 2021;9(2):e17573. DOI: 10.2196/17573](https://www.zotero.org/google-docs/?broken=h3UXok)

[9. Holmen H, Torbjørnsen A, Wahl AK, Jenum AK, Småstuen MC, Arsand E, et al. A Mobile Health Intervention for Self-Management and Lifestyle Change for Persons With Type 2 Diabetes, Part 2: One-Year Results From the Norwegian Randomized  Controlled Trial RENEWING HEALTH. JMIR Mhealth Uhealth; 2014;2(4):e57. DOI: 10.2196/mhealth.3882](https://www.zotero.org/google-docs/?broken=m0sjqq)

[10. Wang Y, Li M, Zhao X, Pan X, Lu M, Lu J, et al. Effects of continuous care for patients with type 2 diabetes using mobile health application: A randomised controlled trial. Int J Health Plann Mgmt; 2019 Jul;34(3):1025–35. DOI: 10.1002/hpm.2872](https://www.zotero.org/google-docs/?broken=ob0dOz)

[11. Waki K, Fujita H, Uchimura Y, Omae K, Aramaki E, Kato S, et al. DialBetics: A Novel Smartphone-based Self-management Support System for Type 2 Diabetes Patients. J Diabetes Sci Technol; 2014 Mar;8(2):209–15. DOI: 10.1177/1932296814526495](https://www.zotero.org/google-docs/?broken=SLZq1j)

[12. Bretschneider MP, Klásek J, Karbanová M, Timpel P, Herrmann S, Schwarz PEH. Impact of a Digital Lifestyle Intervention on Diabetes Self-Management: A Pilot Study. Nutrients; 2022;14(9):1810. DOI: 10.3390/nu14091810](https://www.zotero.org/google-docs/?broken=Jwi9yU)

[13. Dugas M, Wang W, Crowley K, Iyer AK, Peeples M, Shomali M, et al. Engagement and Outcomes Associated with Contextual Annotation Features of a Digital Health Solution. J Diabetes Sci Technol; 2022;16(4):804–11. DOI: 10.1177/1932296820976409](https://www.zotero.org/google-docs/?broken=gqvVwX)

[14. Dixon RF, Zisser H, Layne JE, Barleen NA, Miller DP, Moloney DP, et al. A Virtual Type 2 Diabetes Clinic Using Continuous Glucose Monitoring and Endocrinology Visits. J Diabetes Sci Technol; 2020;14(5):908–11. DOI: 10.1177/1932296819888662](https://www.zotero.org/google-docs/?broken=vMD1GY)

[15. Majithia AR, Kusiak CM, Armento Lee A, Colangelo FR, Romanelli RJ, Robertson S, et al. Glycemic Outcomes in Adults With Type 2 Diabetes Participating in a Continuous Glucose Monitor–Driven Virtual Diabetes Clinic: Prospective Trial. J Med Internet Res; 2020 Aug 28;22(8):e21778. DOI: 10.2196/21778](https://www.zotero.org/google-docs/?broken=ieMb2G)

[16. Koot D, Goh PSC, Lim RSM, Tian Y, Yau TY, Tan NC, et al. A Mobile Lifestyle Management Program (GlycoLeap) for People With Type 2 Diabetes: Single-Arm Feasibility Study. JMIR Mhealth Uhealth; 2019;7(5):e12965. DOI: 10.2196/12965](https://www.zotero.org/google-docs/?broken=G27hAe)

[17. Venkatesan A, Zimmermann G, Rawlings K, Ryan C, Voelker L, Edwards C. Improvements in Glycemic Control and Depressive Symptoms Among Adults With Type 2 Diabetes: Retrospective Study. JMIR Form Res; 2023 Jan 13;0:e41880. DOI: 10.2196/41880](https://www.zotero.org/google-docs/?broken=upzDxE)

[18. Zimmermann G, Venkatesan A, Rawlings K, Scahill MD. Improved Glycemic Control With a Digital Health Intervention in Adults With Type 2 Diabetes: Retrospective Study. JMIR Diabetes; 2021;6(2):e28033. DOI: 10.2196/28033](https://www.zotero.org/google-docs/?broken=nNc7Lq)

[19. Kim EK, Kwak SH, Baek S, Lee SL, Jang HC, Park KS, et al. Feasibility of a Patient-Centered, Smartphone-Based, Diabetes Care System: A Pilot Study. Diabetes Metab J; 2016;40(3):192–201. DOI: 10.4093/dmj.2016.40.3.192](https://www.zotero.org/google-docs/?broken=wumOBY)

[20. Berman MA, Guthrie NL, Edwards KL, Appelbaum KJ, Njike VY, Eisenberg DM, et al. Change in Glycemic Control With Use of a Digital Therapeutic in Adults With Type 2 Diabetes: Cohort Study. JMIR Diabetes; 2018;3(1):e4. DOI: 10.2196/diabetes.9591](https://www.zotero.org/google-docs/?broken=AWUNkE)

[21. Batch BC, Spratt SE, Blalock DV, Benditz C, Weiss A, Dolor RJ, et al. General Behavioral Engagement and Changes in Clinical and Cognitive Outcomes of Patients with Type 2 Diabetes Using the Time2Focus Mobile App for Diabetes Education: Pilot Evaluation. J Med Internet Res; 2021;23(1):e17537. DOI: 10.2196/17537](https://www.zotero.org/google-docs/?broken=ZelfJi)

[22. Krishnakumar A, Verma R, Chawla R, Sosale A, Saboo B, Joshi S, et al. Evaluating Glycemic Control in Patients of South Asian Origin With Type 2 Diabetes Using a Digital Therapeutic Platform: Analysis of Real-World Data. J Med Internet Res; 2021;23(3):e17908. DOI: 10.2196/17908](https://www.zotero.org/google-docs/?broken=HR3xFw)
