# Supplementary material for: Effect of Multimodal App-Based Interventions on Glycemic Control in Patients With Type 2 Diabetes: Systematic Review and Meta-Analysis
Source: J Med Internet Res. 2025 Jan 24;27:e54324. doi: 10.2196/54324 (PMC11806272; doi:10.2196/54324)
Supplement: Multimedia Appendix 5 [file jmir_v27i1e54324_app5.docx]

**Table S1.** Characteristics of apps identified from RCTs.

| **nBuddy Diabetes**** Lim et al. (2021) and Lim et al. (2022) | | | | | | |
| --- | --- | --- | --- | --- | --- | --- |
|  |  | **Tracking** | **Education (as app content)** | **Education (from a HCP*)** | **Automated Feedback** | **Individualized Feedback** |
|  | **Nutrition** | x |  |  | x | x  (dietitians) |
|  | **Physical Activity** | x |  |  | x | x  (dietitians) |
|  | **Self-Management** | x  (nutrition, step goals) |  |  |  | x  (dietitians) |
|  | **Metabolic Parameters** | x  (blood glucose, weight) |  |  | x | x  (dietitians) |
|  | **Not specified** |  | x  (videos, content not specified) |  |  | x  (peer support) |
| **SideKickHealth***** Hilmarsdóttir et al. (2021) | | | | | | |
|  |  | **Tracking** | **Education (as app content)** | **Education (from a HCP*)** | **Automated Feedback** | **Individualized Feedback** |
|  | **Nutrition** | x |  |  |  |  |
|  | **Physical Activity** | x |  |  |  |  |
|  | **Self-Management** | x  (goals, self-monitoring, medication) |  |  |  |  |
|  | **Metabolic Parameters** | x  (weight, blood glucose, blood pressure, blood test results) |  |  |  |  |
|  | **Not specified** |  | x  (general guidance on healthy lifestyle, task completion) |  | X  (health points that mount up + motivational messages from first author) |  |
| **LIBIT*** (excercise & dietary management) + Medilarm*** (medication adherance);** Lee et al. (2021) | | | | | | |
|  |  | **Tracking** | **Education (as app content)** | **Education (from a HCP*)** | **Automated Feedback** | **Individualised Feedback** |
|  | **Nutrition** | x |  |  | x |  |
|  | **Physical Activity** | x | x |  | x |  |
|  | **Self-Management** | x  (medication) |  |  | x  (medication reminders) |  |
|  | **Metabolic Parameters** |  |  |  |  |  |
|  | **Not specified** |  |  | x  (general education before start for IG and CG) | x |  |
| **iCareD only**; Lee et al. (2022)** | | | | | | |
|  | **iCareD** only** | | | | | |
|  |  | **Tracking** | **Education (as app content)** | **Education (from a HCP*)** | **Automated Feedback** | **Individualised Feedback** |
|  | **Nutrition** | x |  |  | x |  |
|  | **Physical Activity** | x |  |  | x |  |
|  | **Self-Management** |  |  |  |  |  |
|  | **Metabolic Parameters** | x  (blood glucose) |  |  | x |  |
|  | **Not specified** |  | x |  |  |  |
|  | **iCareD** + physician communication & feedback**** | | | | | |
|  | **Nutrition** | x |  |  | x | x |
|  | **Physical Activity** | x |  |  | x |  |
|  | **Self-Management** |  |  | x |  | x |
|  | **Metabolic Parameters** | x  (blood glucose) |  | x | x | x |
|  | **Not specified** |  | x |  |  |  |
| **Developed for research**; Pamungkas et al. (2022)** | | | | | | |
|  |  | **Tracking** | **Education (as app content)** | **Education (from a HCP*)** | **Automated Feedback** | **Individualised Feedback** |
|  | **Nutrition** |  | x | x  (online coaching) |  |  |
|  | **Physical Activity** |  | x |  |  |  |
|  | **Self-Management** | x | x | x  (online coaching) | x  (empowering messages) | x  (problem sharing, empowerment & emotional support) |
|  | **Metabolic Parameters** | x  (blood glucose) | x |  |  |  |
|  | **Not specified** |  |  | x  (online coaching) | x  (bidirectional chatbot) | x  (zoom/telephone: track progress of self-management) |
| **Switch Application**; Lee et al. (2018) and M.-K. Lee et al. (2021** | | | | | | |
|  |  | **Tracking** | **Education (as app content)** | **Education (from a HCP*)** | **Automated Feedback** | **Individualised Feedback** |
|  | **Nutrition** | x |  |  |  | x |
|  | **Physical Activity** | x |  |  |  |  |
|  | **Self-Management** | x | x |  |  | x |
|  | **Metabolic Parameters** | x | x |  |  | x |
|  | **Not specified** |  |  | x  (recommendations / education through app messages) |  | x |
| **Few Touch Application**; Holmen et al. (2014)** | | | | | | |
|  | **Few Touch Application only**** | | | | | |
|  |  | **Tracking** | **Education (as app content)** | **Education (from a HCP*)** | **Automated Feedback** | **Individualised Feedback** |
|  | **Nutrition** | x |  |  |  |  |
|  | **Physical Activity** | x |  |  |  |  |
|  | **Self-Management** | x  (goals) |  |  |  |  |
|  | **Metabolic Parameters** | x  (blood glucose) |  |  | x  (visualization, trend reports) |  |
|  | **Not specified** |  | x  (diabetes-related general information) |  | x |  |
|  | **Few Touch Application** + Health Counseling**** | | | | | |
|  | **Nutrition** | x |  | x |  |  |
|  | **Physical Activity** | x |  |  |  |  |
|  | **Self-Management** | x  (goals) |  |  |  |  |
|  | **Metabolic Parameters** | x  (blood glucose) |  | x | x  (visualization, trend reports) |  |
|  | **Not specified** |  | x  (diabetes-related general information) | x | x | x  (1x/month for 4 months) |
| **Developed for research**; Wang et al. (2019)** | | | | | | |
|  |  | **Tracking** | **Education (as app content)** | **Education (from a HCP*)** | **Automated Feedback** | **Individualised Feedback** |
|  | **Nutrition** | x | x |  | x  (recommendations) | x |
|  | **Physical Activity** | x | x | x  (individualized workout plans) | x  (intensity warnings) | x |
|  | **Self-Management** | x | x |  |  | x  (medication) |
|  | **Metabolic Parameters** | x  (blood glucose) | x |  | x  (blood glucose) | x  (blood glucose) |
|  | **Not specified** |  |  | x  (hand-held clinic, according to individual needs) | x  (reports and summaries) | x  (online community forum) |
| **DialBetics***; Waki et al. (2014)** | | | | | | |
|  |  | **Tracking** | **Education (as app content)** | **Education (from a HCP*)** | **Automated Feedback** | **Individualised Feedback** |
|  | **Nutrition** | x |  |  | x | x  (feedback on photos of meals) |
|  | **Physical Activity** | x |  |  | x |  |
|  | **Self-Management** |  |  |  | x |  |
|  | **Metabolic Parameters** | x  (weight, blood glucose, blood pressure) |  |  | x | x  (physician interaction, if necessary, i.e. blood pressure too high) |
|  | **Not specified** |  |  |  |  |  |

*HCP = Health Care Professional

** Diabetes-specific

*** not Diabetes-specific

Note: Diabetes-specific = diabetes-focused content in more than 1 feature (i.e. tracking of blood glucose, feedback on blood glucose tracking, diabetes-specific education); only automated feedback on blood glucose tracking is not counted as an extra feature

**Table S2.** Characteristics of apps identified from non-RCTs.

| **Vitadio**; Bretschneider et al. (2022)** | | | | | | |
| --- | --- | --- | --- | --- | --- | --- |
|  |  | **Tracking** | **Education (as app content)** | **Education (from a HCP*)** | **Automated Feedback** | **Individualized Feedback** |
|  | **Nutrition** | x | x |  |  |  |
|  | **Physical Activity** | x | x |  |  |  |
|  | **Self-Management** | x  (mood) | x  (sleep, mental, social aspects of life with diabetes) |  |  |  |
|  | **Metabolic Parameters** | x  (weight, waist circumference, HbA1c) |  |  |  |  |
|  | **Not specified** |  |  |  | x | x  (advisor and peer support) |
| **BlueStar***; Dugas et al. (2022)** | | | | | | |
|  |  | **Tracking** | **Education (as app content)** | **Education (from a HCP*)** | **Automated Feedback** | **Individualized Feedback** |
|  | **Nutrition** | x |  |  |  |  |
|  | **Physical Activity** | x |  |  |  |  |
|  | **Self-Management** | x  (medication) |  |  |  |  |
|  | **Metabolic Parameters** | x  (blood glucose) |  |  |  |  |
|  | **Not specified** | x  (free text annotations) |  |  | x |  |
| **Onduo Virtual Diabetes Clinic**; Dixon et al. (2020) and Majithia et al. (2020)** | | | | | | |
|  |  | **Tracking** | **Education (as app content)** | **Education (from a HCP*)** | **Automated Feedback** | **Individualised Feedback** |
|  | **Nutrition** | x |  |  |  | x |
|  | **Physical Activity** | x |  |  |  | x |
|  | **Self-Management** | x  (medication) | x | x |  | x |
|  | **Metabolic Parameters** | x  (blood glucose) |  | x |  | x |
|  | **Not specified** |  |  | x | x  (reminders) | x  (lifestyle coaching, consultations with providers via (video) call) |
| **GlykoLeap**; Koot et al. (2019)** | | | | | | |
|  |  | **Tracking** | **Education (as app content)** | **Education (from a HCP*)** | **Automated Feedback** | **Individualised Feedback** |
|  | **Nutrition** | x |  |  | x |  |
|  | **Physical Activity** | x |  |  |  |  |
|  | **Self-Management** |  | x |  |  |  |
|  | **Metabolic Parameters** | x  (weight, blood glucose) | x |  |  |  |
|  | **Not specified** |  |  |  |  | x  (health coach contact) |
| **Vida Health**; Zimmermann et al. (2021) and Venkatesan et al. (2023)** | | | | | | |
|  |  | **Tracking** | **Education (as app content)** | **Education (from a HCP*)** | **Automated Feedback** | **Individualised Feedback** |
|  | **Nutrition** | x | x |  | x |  |
|  | **Physical Activity** | x |  |  |  |  |
|  | **Self-Management** |  | x  (medication) | x  (remote coaching sessions) |  |  |
|  | **Metabolic Parameters** | x  (blood glucose) | x  (blood glucose) |  |  |  |
|  | **Not specified** |  |  |  |  | x |
| **N/A***; Kim et al. (2016)** | | | | | | |
|  |  | **Tracking** | **Education (as app content)** | **Education (from a HCP*)** | **Automated Feedback** | **Individualized Feedback** |
|  | **Nutrition** | x |  |  | x |  |
|  | **Physical Activity** | x | x |  | x |  |
|  | **Self-Management** | x | x |  |  |  |
|  | **Metabolic Parameters** | x  (weight, blood glucose) |  |  | x |  |
|  | **Not specified** |  |  |  |  | x  (peer support) |
| **FareWell***; Berman et al. (2018)** | | | | | | |
|  |  | **Tracking** | **Education (as app content)** | **Education (from a HCP*)** | **Automated Feedback** | **Individualized Feedback** |
|  | **Nutrition** | x | x | x  (phone call) | x |  |
|  | **Physical Activity** | x | x | x  (phone call) |  |  |
|  | **Self-Management** | x  (planning, goal-setting) |  | x  (phone call) | x  (doctoral appointment reminders) |  |
|  | **Metabolic Parameters** | x  (weight) |  |  |  |  |
|  | **Not specified** |  |  |  |  | x  (peer and expert support, phone calls) |
| **Time2Focus**; Batch et al. (2021)** | | | | | | |
|  |  | **Tracking** | **Education (as app content)** | **Education (from a HCP*)** | **Automated Feedback** | **Individualized Feedback** |
|  | **Nutrition** | x | x |  |  |  |
|  | **Physical Activity** | x | x |  |  |  |
|  | **Self-Management** | x  (goal-setting) | x |  | x  (reminders) |  |
|  | **Metabolic Parameters** | x  (blood glucose) | x  (blood glucose) |  |  |  |
|  | **Not specified** |  |  |  |  |  |
| **Wellthy CARE**; Krishnakumar et al. (2021)** | | | | | | |
|  |  | **Tracking** | **Education (as app content)** | **Education (from a HCP*)** | **Automated Feedback** | **Individualized Feedback** |
|  | **Nutrition** | x | x |  | x |  |
|  | **Physical Activity** | x | x |  | x |  |
|  | **Self-Management** |  | x  (medication adherence, diabetes self-care) |  | x |  |
|  | **Metabolic Parameters** | x  (weight, blood glucose) |  |  |  |  |
|  | **Not specified** |  |  |  |  | x  (coaching calls or messages) |

*HCP = Health Care Professional

** Diabetes-specific

*** not Diabetes-specific

Note: Diabetes-specific = diabetes-focused content in more than 1 feature (i.e. tracking of blood glucose, feedback on blood glucose tracking, diabetes-specific education); only automated feedback on blood glucose tracking is not counted as an extra feature
