# Supplementary material for: Effect of Multimodal App-Based Interventions on Glycemic Control in Patients With Type 2 Diabetes: Systematic Review and Meta-Analysis
Source: J Med Internet Res. 2025 Jan 24;27:e54324. doi: 10.2196/54324 (PMC11806272; doi:10.2196/54324)
Supplement: Multimedia Appendix 6 [file jmir_v27i1e54324_app6.docx]

**ROBINS-I: Risk of Bias Assessment Tool for Non-Randomized Studies**

The ROBINS-I tool by Sterne et al. (2016a) was used to assess the risk of bias for each of the included non-RCTs. The tool breaks down the bias assessment into seven different domains, including selection of participants, classification of exposure, classification of outcomes, and other possible sources of bias. The predicted direction of bias can optionally be answered for each domain with “favors experimental”, “favors comparator”, “towards null”, “away from null”, or “unpredictable”. This was not done in this study. For each domain, the risk of bias risk is rated as "low", "moderate", "serious", or "critical". After the individual assessment in the different domains, the overall risk of bias is determined based on the lowest assessment in one of the domains. The following table explains the interpretation of the overall assessment.

**Table S1.** Interpretation of the overall RoB assessment (Sterne et al., 2016b, p. 18).

| **Judgment** | **Within each domain** | **Across domains** | **Criterion** |
| --- | --- | --- | --- |
| Low RoB | The study is comparable to a well performed randomized trial with regard to this domain. | The study is comparable to a well performed randomized trial. | The study is judged to be at low RoB for all domains. |
| Moderate RoB | The study is sound for a non-randomised study with regard to this domain but cannot be considered comparable to a well performed randomized trial. | The study provides sound evidence for a non-randomised study but cannot be considered comparable to a well performed randomized trial. | The study is judged to be at low or moderate RoB for all domains. |
| Serious RoB | The study has some important problems in this domain. | The study has some important problems. | The study is judged to be at serious RoB in at least one domain, but not at critical RoB in any domain. |
| Critical RoB | The study is too problematic in this domain to provide any useful evidence on the effects of intervention. | The study is too problematic to provide any useful evidence and should not be included in any synthesis. | The study is judged to be at critical RoB in at least one domain. |
| No information | No information on which to base a judgment about RoB for this domain. | No information on which to base a judgment about RoB. | There is no clear indication that the study is at serious or critical RoB and there is a lack of information in one or more key domains of bias (a judgment is required for this). |

RoB = risk of bias

The ROBINS-I tool covers the following seven domains, each consisting of up to eight individual questions that are answered with “yes”, “probably yes”, “no”, “probably no”, or “no information”, respectively. For the present study, time-varying confounding was not applicable, hence the signaling questions 1.7. and 1.8. were not applied for this assessment.

1. **Bias due to confounding**
   1. Is there potential for confounding the effect of intervention in this study?
      If N/PN to 1.1: the study can be considered to be at low risk of bias due to confounding and no further signalling questions need be considered.

If Y/PY to 1.1: determine whether there is a need to assess time-varying confounding:

- 1. Was the analysis based on splitting participants’ follow up time according to intervention received?
     If N/PN, answer questions relating to baseline confounding (1.4 to 1.6) 
     If Y/PY, go to question 1.3.
  2. Were intervention discontinuations or switches likely to be related to factors that are prognostic for the outcome?
     If N/PN, answer questions relating to baseline confounding (1.4 to 1.6)
     If Y/PY, answer questions relating to both baseline and time-varying confounding (1.7 and 1.8)

**Questions relating to baseline confounding only**

- 1. Did the authors use an appropriate analysis method that controlled for all the important confounding domains?
  2. If Y/PY to 1.4: Were confounding domains that were controlled for measured validly and reliably by the variables available in this study?
  3. Did the authors control for any post-intervention variables that could have been affected by the intervention?

**Questions relating to baseline and time-varying confounding**

- 1. Did the authors use an appropriate analysis method that controlled for all the important confounding domains and for time-varying confounding?
  2. If Y/PY to 1.7: Were confounding domains that were controlled for measured validly and reliably by the variables available in this study?

1. **Bias in selection of participants into the study**
   1. Was selection of participants into the study (or into the analysis) based on participant characteristics observed after the start of intervention?
      If N/PN to 2.1: go to 2.4
   2. If Y/PY to 2.1: Were the post-intervention variables that influenced selection likely to be associated with intervention?
   3. If Y/PY to 2.2: Were the post-intervention variables that influenced selection likely to be influenced by the outcome or a cause of the outcome?
   4. Do start of follow-up and start of intervention coincide for most participants?
   5. If Y/PY to 2.2 and 2.3, or N/PN to 2.4: Were adjustment techniques used that are likely to correct for the presence of selection biases?
2. **Bias in classification of interventions**
   1. Were intervention groups clearly defined?
   2. Was the information used to define intervention groups recorded at the start of the intervention?
   3. Could classification of intervention status have been affected by knowledge of the outcome or risk of the outcome?
3. **Bias due to deviations from intended interventions**

**If your aim for this study is to assess the effect of assignment to intervention, answer questions 4.1 and 4.2**

- 1. Were there deviations from the intended intervention beyond what would be expected in usual practice?
  2. If Y/PY to 4.1: Were these deviations from intended intervention unbalanced between groups and likely to have affected the outcome?

**If your aim for this study is to assess the effect of starting and adhering to intervention, answer questions 4.3 to 4.6**

- 1. Were important co-interventions balanced across intervention groups?
  2. Was the intervention implemented successfully for most participants?
  3. Did study participants adhere to the assigned intervention regimen?
  4. If N/PN to 4.3, 4.4 or 4.5: Was an appropriate analysis used to estimate the effect of starting and adhering to the intervention?

1. **Bias due to missing data**
   1. Were outcome data available for all, or nearly all, participants?
   2. Were participants excluded due to missing data on intervention status?
   3. Were participants excluded due to missing data on other variables needed for the analysis?
   4. If PN/N to 5.1, or Y/PY to 5.2 or 5.3: Are the proportion of participants and reasons for missing data similar across interventions?
   5. If PN/N to 5.1, or Y/PY to 5.2 or 5.3: Is there evidence that results were robust to the presence of missing data?
2. **Bias in measurement of outcomes**
   1. Could the outcome measure have been influenced by knowledge of the intervention received?
   2. Were outcome assessors aware of the intervention received by study participants?
   3. Were the methods of outcome assessment comparable across intervention groups?
   4. Were any systematic errors in measurement of the outcome related to intervention received?
3. **Bias in selection of the reported result**
   1. ... multiple outcome measurements within the outcome domain?
   2. ... multiple analyses of the intervention-outcome relationship?
   3. ... different subgroups?

**ROBINS-I: Risk of Bias Assessment of Studies Included**

| ID | CO* | 1.1. | 1.2.  ** | 1.4. | 1.5. | 1.6. | **ROB** | 2.1. | 2.2. | 2.3. | 2.4. | 2.5. | **ROB** | 3.1. | 3.2. | 3.3. | **ROB** | 4.1. | 4.2. | **ROB** |
| --- | --- | --- | --- | --- | --- | --- | --- | --- | --- | --- | --- | --- | --- | --- | --- | --- | --- | --- | --- | --- |
| 658 | 1, 2, 3, 4 | Y | N | N |  | N | S | N |  |  | Y |  | L | Y | Y | N | L | PN |  | L |
| 649 | 1, 2, 3 | Y | N | N |  | N | S | N |  |  | Y |  | L | Y | Y | N | L | PN |  | L |
| 73 | 1, 2, 3, 4, 5 | Y | N | N |  | N | S | Y | PY | Y | PN | N | C | Y | Y | N | L | PN |  | L |
| 191 | 1, 3 | Y | N | N |  | N | S | Y | PY | Y | N | N | C | Y | Y | N | L | PN |  | L |
| 484 | 1, 3 | Y | N | N |  | N | S | Y | PY | Y | N | N | C | Y | Y | N | L | PN |  | L |
| 237 | 1, 2, 3, 4* | Y | N | N |  | N | S | N |  |  | Y |  | L | Y | Y | N | L | PN |  | L |
| 140 | 1, 2, 3, 4 | Y | N | N |  | N | S | N |  |  | Y |  | L | Y | Y | N | L | PN |  | L |
| 54 | 1, 2, 6 | Y | N | N |  | N | S | N |  |  | Y |  | L | Y | Y | N | L | PN |  | L |
| 646 | 1*, 2, 3 | Y | N | Y | Y | N | M | PN |  |  | NI |  | NI | Y | Y | N | L | PN |  | L |
| 317 | 1*, 4*, 5*, 6*, 7* | Y | N | Y | Y | Y | S | N |  |  | PN | N | S | Y | Y | N | L | PN |  | L |

|  | 5.1. | 5.2. | 5.3. | 5.4. | 5.5. | **ROB** | 6.1. | 6.2. | 6.3. | 6.4. | **ROB** | 7.1. | 7.2. | 7.3. | **ROB** | **ROB (Overall)** |
| --- | --- | --- | --- | --- | --- | --- | --- | --- | --- | --- | --- | --- | --- | --- | --- | --- |
| 658 | Y | N | N |  |  | L | N | PY | Y | N | L | N | PN | PN | M | S |
| 649 | N | N | N |  | N | C | N | PY | Y | N | L | N | PN | PN | M | C |
| 73 | N | N | PN |  | N | C | N | Y | PN | N | S | N | PN | N | M | C |
| 191 | N | N | N |  | N | C | N | PN | PY | PN | L | N | N | N | M | C |
| 484 | N | N | N |  | N | C | N | PN | PY | PN | L | N | N | N | M | C |
| 237 | Y | N | N |  |  | L | N | Y | Y | PN | L | N | N | N | M | S |
| 140 | Y | N | N |  |  | L | N | PY | Y | N | L | N | N | N | M | S |
| 54 | N | N | N | PN | N | C | N | PY | Y | N | L | N | PN | PN | M | C |
| 646 | NI | N | NI |  |  | NI | N | N | Y | N | L | N | PN | PN | M | NI |
| 317 | Y | N | N |  |  | L | N | Y | PN | N | S | N | N | N | M | S |

CO: confounders (1: HbA1c; 2: age; 3: gender, 4: medication, 5: app-engagement , 6: diabetes duration, 7: BMI)

L: low; M: moderate; S: serious; C: critical

RoB: risk of bias

Y: yes; PY: probably yes; N: no; PN: probably no; NI: no information

* adjusted for in study

** always no, because of the nature of the intervention

**Literature**

| a | Sterne JAC, Hernán MA, Reeves BC, Savović J, Berkman ND, Viswanathan M, Henry D, Altman DG, Ansari MT, Boutron I, Carpenter JR, Chan AW, Churchill R, Deeks JJ, Hróbjartsson A, Kirkham J, Jüni P, Loke YK, Pigott TD, Ramsay CR, Regidor D, Rothstein HR, Sandhu L, Santaguida PL, Schünemann HJ, Shea B, Shrier I, Tugwell P, Turner L, Valentine JC, Waddington H, Waters E, Wells GA, Whiting PF, Higgins JPT. ROBINS-I: a tool for assessing risk of bias in non-randomized studies of interventions. BMJ 2016; 355; i4919. |
| --- | --- |
| b | Sterne JAC, Higgins JPT, Elbers RG, Reeves BC and the development group for ROBINS-I. Risk Of Bias In Non-randomized Studies of Interventions (ROBINS-I): detailed guidance, updated 12 October 2016. Available from http://www.riskofbias.info [accessed 19.09.2023] |
| 646 | Batch BC, Spratt SE, Blalock DV, et al. General Behavioral Engagement and Changes in Clinical and Cognitive Outcomes of Patients with Type 2 Diabetes Using the Time2Focus Mobile App for Diabetes Education: Pilot Evaluation. J Med Internet Res. 2021;23(1):e17537. doi:10.2196/17537 |
| 317 | Berman MA, Guthrie NL, Edwards KL, et al. Change in Glycemic Control With Use of a Digital Therapeutic in Adults With Type 2 Diabetes: Cohort Study. JMIR Diabetes. 2018;3(1):e4. doi:10.2196/diabetes.9591 |
| 54 | Bretschneider MP, Klásek J, Karbanová M, Timpel P, Herrmann S, Schwarz PEH. Impact of a Digital Lifestyle Intervention on Diabetes Self-Management: A Pilot Study. Nutrients. 2022;14(9). doi:10.3390/nu14091810 |
| 73 | Dugas M, Wang W, Crowley K, et al. Engagement and Outcomes Associated with Contextual Annotation Features of a Digital Health Solution. J Diabetes Sci Technol. 2022;16(4):804-811. doi:10.1177/1932296820976409 |
| 237 | Kim EK, Kwak SH, Baek S, et al. Feasibility of a Patient-Centered, Smartphone-Based, Diabetes Care System: A Pilot Study. Diabetes Metab J. 2016;40(3):192-201. doi:10.4093/dmj.2016.40.3.192 |
| 140 | Koot D, Goh PSC, Lim RSM, et al. A Mobile Lifestyle Management Program (GlycoLeap) for People With Type 2 Diabetes: Single-Arm Feasibility Study. JMIR Mhealth Uhealth. 2019;7(5):e12965. doi:10.2196/12965 |
| 649 | Krishnakumar A, Verma R, Chawla R, et al. Evaluating Glycemic Control in Patients of South Asian Origin With Type 2 Diabetes Using a Digital Therapeutic Platform: Analysis of Real-World Data. J Med Internet Res. 2021;23(3):e17908. doi:10.2196/17908 |
| 658 | Majithia AR, Kusiak CM, Armento Lee A, et al. Glycemic Outcomes in Adults With Type 2 Diabetes Participating in a Continuous Glucose Monitor–Driven Virtual Diabetes Clinic: Prospective Trial. J Med Internet Res. 2020;22(8):e21778. doi:10.2196/21778 |
| 484 | Venkatesan A, Zimmermann G, Rawlings K, Ryan C, Voelker L, Edwards C. Improvements in Glycemic Control and Depressive Symptoms Among Adults With Type 2 Diabetes: Retrospective Study. JMIR Form Res. 2023;0:e41880. doi:10.2196/41880 |
| 191 | Zimmermann G, Venkatesan A, Rawlings K, Scahill MD. Improved Glycemic Control With a Digital Health Intervention in Adults With Type 2 Diabetes: Retrospective Study. JMIR Diabetes. 2021;6(2):e28033. doi:10.2196/28033 |
