# Supplementary material for: Effect of Multimodal App-Based Interventions on Glycemic Control in Patients With Type 2 Diabetes: Systematic Review and Meta-Analysis
Source: J Med Internet Res. 2025 Jan 24;27:e54324. doi: 10.2196/54324 (PMC11806272; doi:10.2196/54324)
Supplement: Multimedia Appendix 7 [file jmir_v27i1e54324_app7.docx]

**Table S1.** Outlier analysis between-group differences.

| Analysis | MD | 95%CI | p | I2 | 95%CI |
| --- | --- | --- | --- | --- | --- |
| all cases | -0.36 | [-0.59; -0.14] | 0.008 | 19.1% | [0.0%; 60.8%] |
| Infl. Cases Removed^a^ | -0.33 | [-0.56; -0.10] | 0.010 | 41.3% | [0.0%; 71.0%] |

^a^[Zimmermann et al., 2021](https://www.zotero.org/google-docs/?WdjK3q)

**Table S2.** Outlier analysis within-group differences.

| Analysis | MD | 95%CI | p | I2 | 95%CI |
| --- | --- | --- | --- | --- | --- |
| all cases | -0.79 | [-1.01; -0.55] | < 0.001 | 91.8% | [88.7%; 94.1] |
| Infl. Cases Removed^a^ | -0.69 | [-0.84; -0.54] | < 0.001 | 61.1% | [31.8%; 77.9%] |

^a^[Lee et al., 2021; Majithia et al., 2020; Pamungkas et al., 2022; Venkatesan et al., 2023](https://www.zotero.org/google-docs/?DTnfHw)

**Literature**

1. [Lee SE, Park SK, Park YS, Kim KA, Choi HS, Oh SW. Effects of Short-term Mobile Application Use on Weight Reduction for Patients with Type 2 Diabetes. J Obes Metab Syndr; 2021;30(4):345–53. DOI: 10.7570/jomes21047](https://www.zotero.org/google-docs/?broken=md8rJS)

2. [Majithia AR, Kusiak CM, Armento Lee A, Colangelo FR, Romanelli RJ, Robertson S, et al. Glycemic Outcomes in Adults With Type 2 Diabetes Participating in a Continuous Glucose Monitor–Driven Virtual Diabetes Clinic: Prospective Trial. J Med Internet Res; 2020 Aug 28;22(8):e21778. DOI: 10.2196/21778](https://www.zotero.org/google-docs/?broken=ieMb2G)

3. [Pamungkas RA, Usman AM, Chamroonsawasdi K, Abdurrasyid. A smartphone application of diabetes coaching intervention to prevent the onset of complications and to improve diabetes self-management: A randomized control  trial. Diabetes Metab Syndr; 2022;16(7):102537. DOI: 10.1016/j.dsx.2022.102537](https://www.zotero.org/google-docs/?broken=I9tb7d)

4. [Venkatesan A, Zimmermann G, Rawlings K, Ryan C, Voelker L, Edwards C. Improvements in Glycemic Control and Depressive Symptoms Among Adults With Type 2 Diabetes: Retrospective Study. JMIR Form Res; 2023 Jan 13;0:e41880. DOI: 10.2196/41880](https://www.zotero.org/google-docs/?broken=upzDxE)

5. [Zimmermann G, Venkatesan A, Rawlings K, Scahill MD. Improved Glycemic Control With a Digital Health Intervention in Adults With Type 2 Diabetes: Retrospective Study. JMIR Diabetes; 2021;6(2):e28033. DOI: 10.2196/28033](https://www.zotero.org/google-docs/?broken=nNc7Lq)
